# Genome-wide identification of the *CAD* gene family and functional analysis of putative *bona fide CAD* genes in tobacco (*Nicotiana tabacum* L.)

**DOI:** 10.3389/fpls.2024.1400213

**Published:** 2024-07-08

**Authors:** Mingzhu Wu, Yijun Li, Zhengtai Liu, Lin Xia, Yiyu Xiang, Lijie Zhao, Xiaobei Yang, Zefeng Li, Xiaodong Xie, Lin Wang, Ren Wang, Sheng Xu, Jun Yang

**Affiliations:** ^1^ China Tobacco Gene Research Center, Zhengzhou Tobacco Research Institute of China National Tobacco Corporation (CNTC), Zhengzhou, China; ^2^ Nanjing University of Chinese Medicine, Nanjing, China; ^3^ Institute of Botany, Jiangsu Province and Chinese Academy of Sciences, Nanjing, China; ^4^ College of Life Science, Henan Agricultural University, Zhengzhou, China

**Keywords:** cinnamyl alcohol dehydrogenase, lignin, enzymatic assay, stresses, tobacco

## Abstract

Cinnamyl alcohol dehydrogenase (CAD) plays a crucial role in lignin biosynthesis, and the gene family encoding various CAD isozymes has been cloned and characterized in numerous plant species. However, limited information regarding the *CAD* gene family in tobacco is currently available. In this study, we identified 10 *CAD* genes in *Nicotiana tabacum*, four in *N. tomentosiformis*, and six in *N. sylvestris*. The nucleotide and amino acid sequences of these tobacco CADs demonstrate high levels of similarity, whereas the putative protein sequences conservatively possessed two Zn^2+^ binding motifs and an NADP(H) cofactor binding motif. Both NtCAD1 and NtCAD2 had conservative substrate binding sites, similar to those possessed by *bona fide* CADs, and evidence from phylogenetic analysis as well as expression profiling supported their role as *bona fide* CADs involved in lignin biosynthesis. NtCAD1 has two paralogous genes, *NtCAD1–1* and *NtCAD1–2*. Enzyme activity analysis revealed that NtCAD1–1 and NtCAD1–2 had a high affinity to coniferyl aldehyde, *p*-coumaryl aldehyde, and sinapyl aldehyde, whereas NtCAD2 preferred coniferyl aldehyde and *p*-coumaryl aldehyde as substrates. The kinetic parameter assay revealed that NtCAD1–2 functions as the most efficient enzyme. Downregulation of both NtCAD1–1 and NtCAD1–2 resulted in reddish-brown stems without significant changes in lignin content. Furthermore, NtCAD1–1, NtCAD1–2, and NtCAD2 showed distinct expression patterns in response to biotic and abiotic stresses, as well as different phytohormones. Our findings suggest that NtCAD1–1 and NtCAD1–2 are involved in lignin biosynthesis, with NtCAD1–2 also participating in both biological and abiotic stresses, whereas NtCAD2 plays a distinct role mainly in responding to biological and abiotic stresses in tobacco.

## 1 Introduction

Lignin, a vital structural component of plant cell walls, plays a pivotal role in plant development, water transport, mechanical support of the cell wall, defense against pathogens and insects, and enhancement of resistance to abiotic stress (Cesarino, 2019). Lignin synthesis is a complex process involving numerous enzymes and intermediates (Liu et al., 2018; Vanholme et al., 2019). Cinnamyl alcohol dehydrogenase (CAD, EC 1.1.1.195), discovered in 1973 (Gross et al., 1973), is an NADPH-dependent enzyme that catalyzes the final step in the reduction of three hydroxycinnamaldehydes (sinapyl aldehyde, *p*-coumaryl aldehyde, and coniferyl aldehyde) to their corresponding hydroxycinnamyl alcohols, with zinc acting as a cofactor (O’malley et al., 1992; Kim et al., 2004). This enzymatic reaction is indispensable for generating monomeric lignin precursors and contributes significantly to the diversity of lignin compositions. The primary constituents of aromatic lignin polymers commonly observed in angiosperms are sinapyl alcohols, *p*-coumaryl alcohols, and coniferyl alcohols. These monolignols undergo polymerization to yield syringyl (S), *p*-hydroxyphenyl (H), and guaiacyl (G) lignin (Li et al., 2008a). CAD and its homologs have been identified in multigene families of various plant species. Specifically, melon and mulberry each have 5 members (Jin et al., 2014; Chao et al., 2022), *Brachypodium distachyon* and oil palm each have 7 members (Bukh et al., 2012; Yusuf et al., 2022), *Arabidopsis* and poplar each have 9 members (Kim et al., 2004; Barakat et al., 2009; Chao et al., 2014), rice has 12 members (Tobias and Chow, 2005), sorghum has 14 members (Saballos et al., 2009), and pomegranate has 25 members (Hu et al., 2022).

*CAD*-deficient plants display a brown midrib phenotype, characterized by a distinct reddish-brown color and altered lignin biosynthesis. This phenotypic variation has been observed in several plant species, including rice (Zhang et al., 2006), maize (Halpin et al., 1998; Kolkman et al., 2023), sorghum (Sattler et al., 2009; Rivera-Burgos et al., 2019), poplar (Baucher et al., 1996), and mulberry (Chao et al., 2022). This change in stem color is attributed to the accumulation or enrichment of coniferyl aldehydes (Li et al., 2008b). Downregulation of *CAD* expression has varying effects on lignin content across different plant species. For example, in a maize *bm1* mutant with a mutation in the *ZmCAD2* regulator, there was a 10%–20% reduction in lignin content, along with a decrease in the S/G ratio (Guillaumie et al., 2007). Similarly, transgenic tall fescue plants with downregulated *CAD* expression exhibited a 28% decrease in lignin content and S/G ratio compared with control plants (Chen et al., 2003). In alfalfa, decreased activity of the CAD enzyme resulted in a lower S/G ratio whereas lignin content remained unchanged (Baucher et al., 1999). Similarly, transgenic *Arabidopsis* and tobacco plants with reduced *CAD* expression showed only a slight decrease in lignin content (Bernard Vailhé et al., 1998; Kim et al., 2004).

CADs in angiosperm species are classified into distinct classes based on phylogenetic tree analysis (Ma, 2010; Bukh et al., 2012; Jin et al., 2014; Hu et al., 2022; Yusuf et al., 2022). Comprehensive evolutionary analyses of CADs from diverse plant species have unequivocally revealed the involvement of *bona fide CAD* genes in monolignol biosynthesis (Guo et al., 2010). Notably, AtCAD4 and AtCAD5 of *Arabidopsis* (Kim et al., 2004), EgCAD2 of Eucalyptus (Goffner et al., 1992), OsCAD2 of rice (Hirano et al., 2012), and ZmCAD2 of *Zea mays* (Liu et al., 2021) play key roles in this process. Furthermore, other CAD homologs appear to play important roles in plant defense against both abiotic and biotic stresses (Barakat et al., 2010; Kim et al., 2010; Park et al., 2018).

Tobacco is an important crop that serves as both a model plant for scientific research and an economic crop. Individual *CAD* genes in tobacco have been extensively characterized in terms of mutant phenotypes, encoded protein activity, and lignin content (Halpin et al., 1992; Chabannes et al., 2001; Damiani et al., 2005; Weiller et al., 2020). However, studies on the expression patterns of *NtCAD* genes in tobacco remain limited. With the completion of tobacco genome sequencing, it now offers great convenience for the comprehensive analysis of the *CAD* gene family at a genomic scale. In this study, we identified 20 putative *CAD* genes from allotetraploid tobacco (*N. tabacum*) and two diploid tobacco species (*N. tomentosiformis* and *N. sylvestris*). Simultaneously, we investigated the genomic location, intron/exon organization, evolutionary relationships among members of the tobacco *CAD* gene family, and expression patterns across different tissues. Furthermore, systematic functional analysis was conducted on three putative *bona fide* NtCADs from *N. tabacum*. The results presented here provide *in vivo* and *in vitro* evidence to elucidate the function of *bona fide CAD* genes in regulating monolignol composition and stress responses in tobacco plants.

## 2 Materials and methods

### 2.1 Plant materials and growth conditions

The tobacco cultivar K326 (*N. tabacum*) obtained from the Yunnan Academy of Tobacco Agricultural Sciences (Kunming, China) was used in all experiments unless otherwise specified. K326 seeds were subjected to surface sterilization with 10% NaClO for 10 min, followed by thorough rinsing with distilled water multiple times. Subsequently, the seeds were germinated and cultivated in plastic pots under standard conditions (daily: 28 ± 1°C for 16 h under 200 μmol m^−2^ s^−1^ light, followed by 23 ± 1°C for 8 h of darkness) until reaching a stage with five to six true leaves (approximately 2 months old). Afterward, they were transplanted into open fields and grown for another 2 months. Then, various plant tissues, including young (leaf number 15, counted from bottom) and senescent (leaf number 5, counted from bottom) tobacco leaves, the veins of the young and senescent leaves, lateral and fibrous roots, stems (from leaf number 9 to leaf number 10, counted from bottom), and axillary and flower buds, were collected for RNA extraction at the flowering stage.

Four-week-old tobacco seedlings (with three to five leaves) were subjected to various abiotic and biotic stresses under identical growth conditions. These stresses included cold treatment (4°C, 24 h), heat treatment (40°C, 2 h), a 10-day drought stress, and a 5-day dark treatment. The conidia of *E. cichoracearum* DC., extracted from four severely infected tobacco leaves using 10 mL of sterile distilled water containing one drop of Tween 20 (Sigma-Aldrich, St. Louis, USA), were subsequently inoculated onto the tobacco seedlings using the spray method. After the 10-day treatment, leaves were collected from the seedlings for further analysis. For abscisic acid (ABA), methyl jasmonate (MeJA), and salicylic acid (SA) treatments, the roots of the seedlings were immersed in solutions of 10 μM ABA, 50 μM MeJA, or 100 μM SA in 1/2 Hoagland solution for 2 h. As a control, seedlings were treated with only 1/2 Hoagland solution.

### 2.2 Phylogenetic and gene structure analyses

CAD sequences of other plant species were retrieved from the NCBI GenBank database using the keyword “cinnamyl alcohol dehydrogenase” as the query. DNAMAN (version 6.0) and ClustalX (version 1.83) were used for multiple alignments of *CAD* nucleotide and deduced amino acid sequences, respectively, with default gap penalties. A phylogenetic tree of CAD amino acid sequences was constructed using MEGA 7.0 with a neighbor-joining algorithm.

### 2.3 RNA extraction and cDNA synthesis

The SuperPure Plant Poly RNA Kit (Codon Biotechnology Co., Beijing, China) was used to extract total RNA from each biological sample, following the manufacturer’s instructions. RNase-free DNase I (TaKaRa Bio Inc., Dalian, China) was used to eliminate DNA contamination during the extraction process. A NanoDrop 2000 instrument (Thermo Fisher Scientific, USA) was used to assess the quality and concentration of extracted RNA. For first-strand cDNA synthesis, 1 μg of total RNA was subjected to PrimeScript™ RT Reagent Kit (TaKaRa, Bio Inc., Dalian, China) with random primers.

### 2.4 Quantitative real-time PCR analysis

Gene expression levels in each sample were measured using quantitative real-time PCR (qRT-PCR) with the TB Green^®^ Premix Ex Taq™ II Reagent (TaKaRa, Bio Inc., Dalian, China) and analyzed using a CFX96 Touch Real-Time PCR System (Bio-Rad, USA). Gene-specific primers were designed and synthesized (**Supplementary Table 1**), and the reference gene *L25* was used for normalization (Schmidt and Delaney, 2010). Relative expression levels of each gene in treated tobacco samples were determined by comparison with their corresponding control samples at specific time points or under specific conditions after normalization to *L25* transcript levels according to 2^−ΔΔCt^ method.

### 2.5 Cloning of tobacco *bona fide CAD* genes

PCR was used to amplify the open reading frames (ORFs) of NtCAD1–1, NtCAD1–2, and NtCAD2 from root tissue cDNA. Gene-specific primers (**Supplementary Table 3**) were employed for amplification, and a three-step program with a gradient annealing temperature ranging from 50°C to 60°C was utilized. The PCR products were purified by gel electrophoresis and cloned into the pMD-19T vector (TaKaRa Bio Inc., Dalian, China) for sequencing (Liuhe Huada Gene Technology Co., Ltd., Beijing, China).

### 2.6 Prokaryotic expression and purification of NtCADs

NtCAD1–1, NtCAD1–2, and NtCAD2 were cloned into the pET-28a (+) plasmid containing 6×His-tagged proteins using the ClonExpress Ultra One Step Cloning Kit (Vazyme Biotech Co., Ltd., Nanjing, China). After sequence confirmation, E. coli BL21 (DE3) cells were transformed with the resulting plasmids: pET-28(a)-NtCAD1–1, pET-28(a)-NtCAD1–2, and pET-28(a)-NtCAD2. The transformed cells were then cultured in LB media supplemented with kanamycin until reaching an optical density at 600 nm (OD600) of approximately 0.6 to 0.8 at 37°C. Protein expression was induced by adding isopropyl-β-D-thiogalactopyranoside (IPTG) to a final concentration of 0.2 mM and incubating at 15°C for 12 h. Cells were then harvested through centrifugation at 4,000 g and 4°C for 15 min and stored at −20°C until further use. His-tagged NtCADs were purified using a previously published method (Sun et al., 2018), followed by SDS-PAGE analysis and quantification using the Bradford Protein Assay Kit (Yeasen Biotechnology Co., Ltd., Shanghai, China).

### 2.7 Measurement of NtCAD activity

Enzymatic activity assays and kinetic determinations were performed according to established methods described in previous studies (O’malley et al., 1992; Chao et al., 2014). NtCAD1–1, NtCAD1–2, and NtCAD2 catalyzed three different substrates (coniferyl aldehyde, *p*-coumaryl aldehyde, and sinapyl aldehyde, respectively) obtained from Yuanye Biotechnology Co., Ltd. (Shanghai, China). The reduction in substrate concentration was measured using a SpectraMax Plus Microplate Reader (Molecular Devices, Shanghai, China). *K_m_* and *V_max_* values were determined by fitting the experimental data to the Michaelis–Menten equation using OriginPro 8.5 software (OriginLab Corporation, USA).

### 2.8 Structural modeling and docking analysis of NtCADs

The NtCAD1–1, NtCAD1–2, and NtCAD2 sequences were analyzed using SWISS-MODEL to search for templates and model their 3D structures, following established methods (Waterhouse et al., 2018). The crystal structure of AtCAD5 was used as the template to create the model. Subsequently, we obtained the 3D structures of coniferyl aldehyde and *p*-coumaryl aldehyde from the PubChem website (https://pubchem.ncbi.nlm.nih.gov/). The PyMOL software was utilized for processing protein, including removing water molecules and adding hydrogen atoms. PyRx-0.8 (https://sourceforge.net/projects/pyrx/) was employed for molecular docking. Discovery Studio 4.5 (Accelrys Inc., San Diego, CA, USA) was used for the visualization of the 3D models and molecular docking structures.

### 2.9 Obtaining *NtCAD1* and *NtCAD2* VIGS transgenic tobacco

Transgenic tobacco plants with reduced *NtCAD1–1*, *NtCAD1–2*, and *NtCAD2* expression were created using virus-induced gene silencing (VIGS). The In-Fusion^®^ Snap Assembly Master Mix from TaKaRa Bio Inc., Dalian, China, was used to insert interference fragments that target *NtCAD1–1*, *NtCAD1–2*, and *NtCAD2* into the pTRV2 vector. All primers used to construct the plasmids contained specific sequences corresponding to the genes of interest, along with a 20-bp recombination arm sequence derived from pTRV2 (**Supplementary Table 2**). Following purification of the fragments and Pst I digestion of the pTRV2 plasmids, ligation was performed using an infusion enzyme, according to the manufacturer’s instructions. After ligation, the products were transformed into *E. coli* DH5α and confirmed by sequencing analysis. The recombinant plasmids pTRV2-NtCAD1–1, pTRV2-NtCAD1–1, pTRV2-NtCAD2, pTRV1, and pTRV2 (negative control) were transformed into *Agrobacterium tumefaciens* LBA4404 using the freeze-thaw method, as described previously (Cowan et al., 2023). The knockdown efficiencies of the three NtCADs were evaluated using qRT-PCR, and their expression levels were compared with those of the control group.

### 2.10 Determination of lignin content

Lignin content was determined using a Lignin Content Assay Kit (Sangon Biotech Co., Ltd., Shanghai, China), following the manufacturer’s instructions. The determination of *p*-hydroxyphenyl (H), guaiacyl (G), and syringyl (S) lignin-derived monomers was carried out using established methods from previous studies (Besseau et al., 2007) by using a Thermo gas chromatograph-mass spectrometry (GC-MS) technology (TRACE 1310-ISQ, Thermo, USA). A Phloroglucinol assay kit (Yuanye Biotechnology Co., Ltd., Shanghai, China) was used to stain the cross-sectioned stems, which were obtained from the reddish-brown part of the stems or from the same part of other treatments of tobacco (*Nicotiana benthamiana*), in accordance with the manufacturer’s instructions. Microscopic examination of the stained sections was performed using a Leica CH-9435 microscope (Heerbrugg, Germany), and a digital camera was used for image capture.

### 2.11 Statistical analysis

The results presented in this study are expressed as the means ± standard deviation (SD) of at least four independent experiments for each treatment. Statistical analysis was conducted using Duncan’s multiple test (*P* < 0.05) or *t*-test (*P* < 0.05 or *P* < 0.01).

## 3 Results

### 3.1 Identification of *CAD* gene family members in tobacco

A search was conducted in the China Tobacco Genome Database v3.0 (unpublished data) using the keyword “cinnamyl alcohol dehydrogenase” as the query to identify putative *CAD* family genes in *N. tabacum*. Subsequently, BLAST searches were conducted using *Arabidopsis* CAD protein sequences as query proteins. After excluding lower-identity sequences, 10 *NtCAD* genes were obtained (**Table 1**); these *NtCAD* genes exhibited variable genomic DNA sizes ranging from 1,808 bp to 19,306 bp, with corresponding cDNA sequence sizes ranging from 945 bp to 1,407 bp. The predicted amino acids ranged from 315 to 469 (molecular weights ranging from 34.29 kDa to 51.81 kDa), with isoelectric point values varying between 7.03 and 8.07. Moreover, we also characterized four and six *CAD* genes in the genomes of *N. tomentosiformis* and *N. sylvestris*, respectively.

**Table 1:** Characteristics of *CAD* genes in tobacco.

	Gene ID	gDNA size (bp)	CDS size (bp)	Number of exons	Amino acid (aa)	Molecular weight (kD)	Isoelectric point
*Nicotiana tabacum*	Ntab0704510	3,069	1,071	4	357	38.99	5.62
	Ntab0152820	1,808	1,065	6	355	38.69	7.47
	Ntab0529780	3,717	1,071	5	357	38.91	6.05
	Ntab0420320	4,381	1,092	5	364	39.62	7.29
	Ntab0336770	2,496	1,080	6	360	39.52	7.43
	Ntab0554550	1,965	1,080	6	360	39.54	7.87
	Ntab0262430	4,304	1,071	5	357	38.80	6.04
	Ntab0181830	5,510	1,080	6	360	39.50	7.31
	Ntab0868330	7,800	1,407	8	469	51.81	8.07
	Ntab0095940	19,306	945	4	315	34.29	7.03
*Nicotiana tomentosiformis*	Ntom0118530	1,234	1,068	7	355	38.48	7.05
	Ntom0224390	1,634	1,095	3	364	39.51	6.59
	Ntom0265640	3,741	1,074	5	357	38.89	5.84
	Ntom0311040	1,965	1,083	6	360	39.54	7.87
*Nicotiana sylvestris*	Nsyl0054440	4,381	1,095	5	364	39.62	7.30
	Nsyl0093350	2,496	1,083	6	360	39.52	7.43
	Nsyl0290350	3,213	1,074	4	357	38.99	5.62
	Nsyl0426890	11,748	1,683	9	560	62.25	4.65
	Nsyl0449860	4,332	1,074	5	357	38.78	6.05
	Nsyl0459450	8,277	1,089	6	362	39.90	7.34

**Figure 1:**
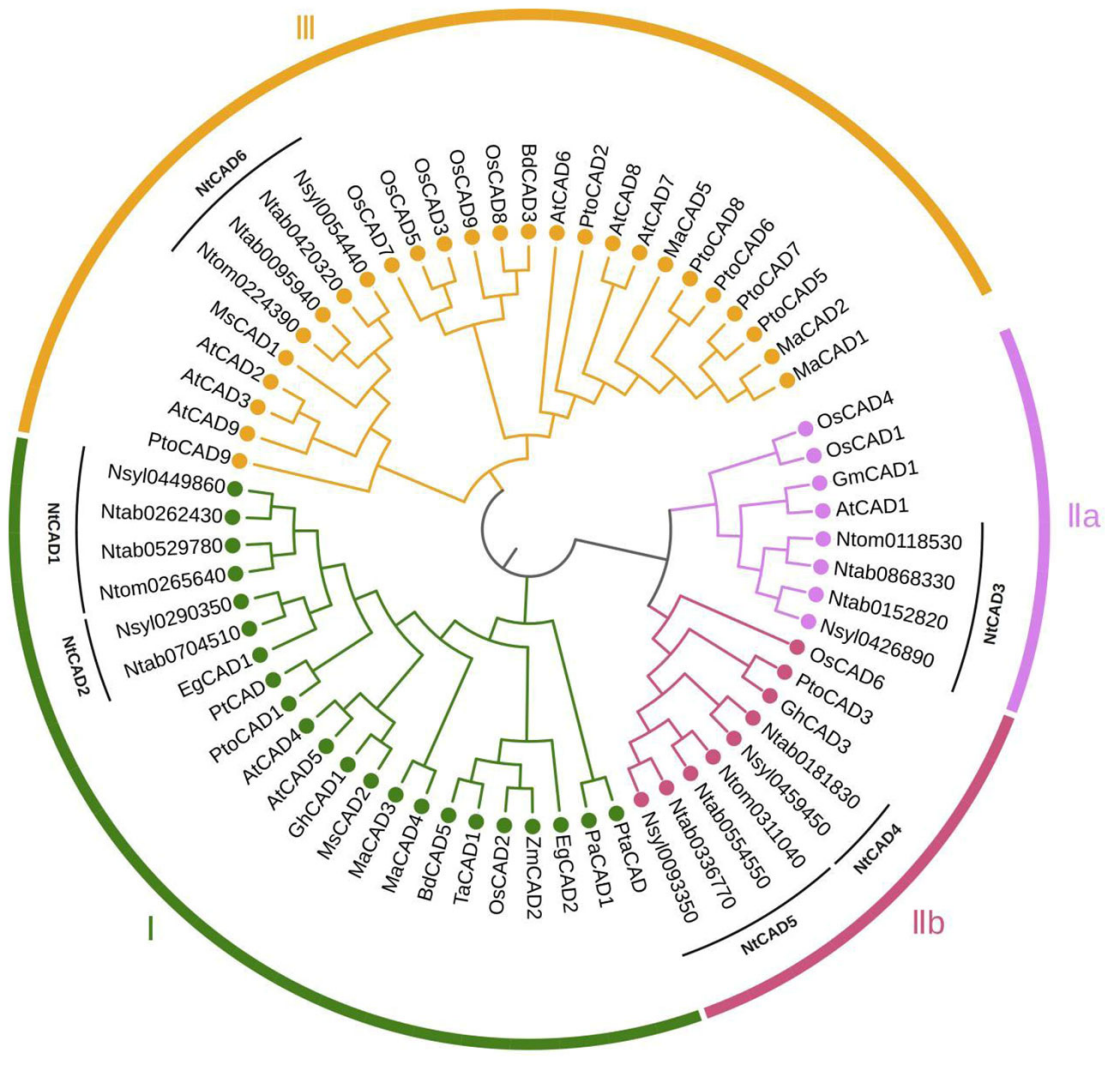
Phylogenetic analysis of tobacco CAD isoforms and other plant CAD homologs. The corresponding plant species and isozymes investigated in this study include tobacco (Ntab, Ntom, and Nsyl); *Arabidopsis thaliana*, AtCAD1–9 (accession number: Q9CAI3, Q9SJ25, Q9SJ10, P48523, O49482, O65621, Q02971, Q02972, P42734); *Oryza sativa*, OsCAD1–9 (Q8H859, Q6ZHS4, Q337Y2, Q2R114, Q0J6T3, Q7XWU3, Q0JA75, Q6ERX1, Q10PS6); *Brachypodium distachyon*, BdCAD3 (AFK80371), BdCAD5 (AFK80372); *Triticum aestivum*, TaCAD1 (GU563724); *Elaeis guineensis*, EgCAD1 (UTE99576), EgCAD (XP_010943210); *Medicago sativa*, MsCAD1 (O82515), MsCAD2 (P31656); *Morus alba*, MaCAD1–5 (UZH97791, UZH97792, UZH97793, UZH97794, UZH97795); *Picea abies*, PaCAD1 (CAA05097); *Populus tremuloides*, PtCAD (AAF43140); *Pinus taeda*, PtaCAD (CAA86073); *Populus tomentosa*, PtoCAD1, 2, 3, 5, 6, 7, 8, 9 (AGU43755, AGU43756, AGU43757, AGU43758, AGU43754, AHX58273, AGU43750, AGU43751); *Zea mays*, ZmCAD2 (CAA74070); *Glycine max*, GmCAD1 (XP_003543132); *Gossypium hirsutum*, GhCAD1 (ABZ01817).

The DNAMAN algorithm was used to calculate the consistency of the 10 *NtCAD* coding and amino acid sequences. As presented in **Table 2**, *NtCAD* genes were classified into four pairs exhibiting high similarity (amino acid sequence identities greater than 72.92%), except Ntab0704510 and Ntab0181830, showing that most of the *NtCAD* genes have two potential paralogs. Furthermore, inter- and intraspecies sequence alignments of NtomCADs and NsylCADs were also performed. It showed that four pairs shared significant levels of sequence identities (60.89%–97.53% at the amino acid level) in interspecies alignment (**Supplementary Table 3**). Subsequently, we designated these 10 *NtCAD* genes in *N. tabacum*, and their putative orthologous genes in *N. sylvestris* and *N. tomentosiformis* (**Table 3**). These results suggest that both diploid genomes possess one ortholog for each *NtCAD* gene, except *NtCAD2* and *NtCAD4* in *N. tomentosiformis*. These homologous relationships were validated by subsequent phylogenetic analyses (**Figure 1**).

**Table 2:** Identity matrix of predicted *NtCAD* genes and their coding amino acid in *N. tabacum*.

		Amino acid identity									
		Ntab0262430	Ntab0529780	Ntab07 04510	Ntab0336770	Ntab0554550	Ntab01 81830	Ntab0420320	Ntab0095940	Ntab08 68330	Ntab0152820
**Nucleotide identity**	Ntab0262430		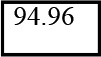	82.91	39.39	39.39	46.11	50.83	43.85	35.38	47.49
	Ntab0529780	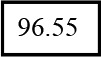		85.43	39.66	39.66	45.66	50.56	43.85	35.17	47.21
	Ntab0704510	83.52	84.54		37.15	37.15	43.33	47.78	41.34	33.05	44.13
	Ntab0336770	55.40	55.49	54.80		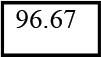	63.06	46.30	45.45	30.87	40.39
	Ntab0554550	56.05	55.77	55.72	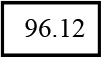		63.33	46.03	45.15	31.08	40.67
	Ntab0181830	56.09	55.35	52.91	76.64	76.92		56.16	47.65	35.10	46.26
	Ntab0420320	55.20	54.52	54.84	59.02	58.83	60.38		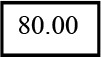	39.71	52.33
	Ntab0095940	49.68	49.12	49.49	51.84	51.75	52.58	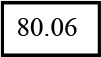		32.91	43.98
	Ntab0868330	42.57	42.99	41.23	42.38	42.45	42.05	43.23	38.06		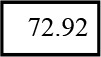
	Ntab0152820	56.55	56.73	53.95	55.57	56.12	55.20	55.92	50.37	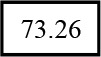	

The values in the boxes indicate a higher level of similarity.

**Table 3:** CAD orthologs among *N. tabacum, N. sylvestris*, and *N. tomentosiformis*.

Protein symbo	Protein symbo	*Nicotiana tabacum*	*Nicotiana sylvestris*	*Nicotiana tomentosiformis*
NtCAD1	NtCAD1–1	Ntab0529780	Nsyl0449860	Ntom0265640
	NtCAD1–2	Ntab0262430		
NtCAD2	NtCAD2	Ntab0704510	Nsyl0290350	NO
NtCAD3	NtCAD3–1	Ntab0868330	Nsyl0426890	Ntom0118530
	NtCAD3–2	Ntab0152820		
NtCAD4	NtCAD4	Ntab0181830	Nsyl0459450	NO
NtCAD5	NtCAD5–1	Ntab0554550	Nsyl0093350	Ntom0311040
	NtCAD5–2	Ntab0336770		
NtCAD6	NtCAD6–1	Ntab0420320	Nsyl0054440	Ntom0224390
	NtCAD6–2	Ntab0095940		

### 3.2 Alignment and chromosomal distributions of tobacco *CADs* across genomes

**Figure 2:**
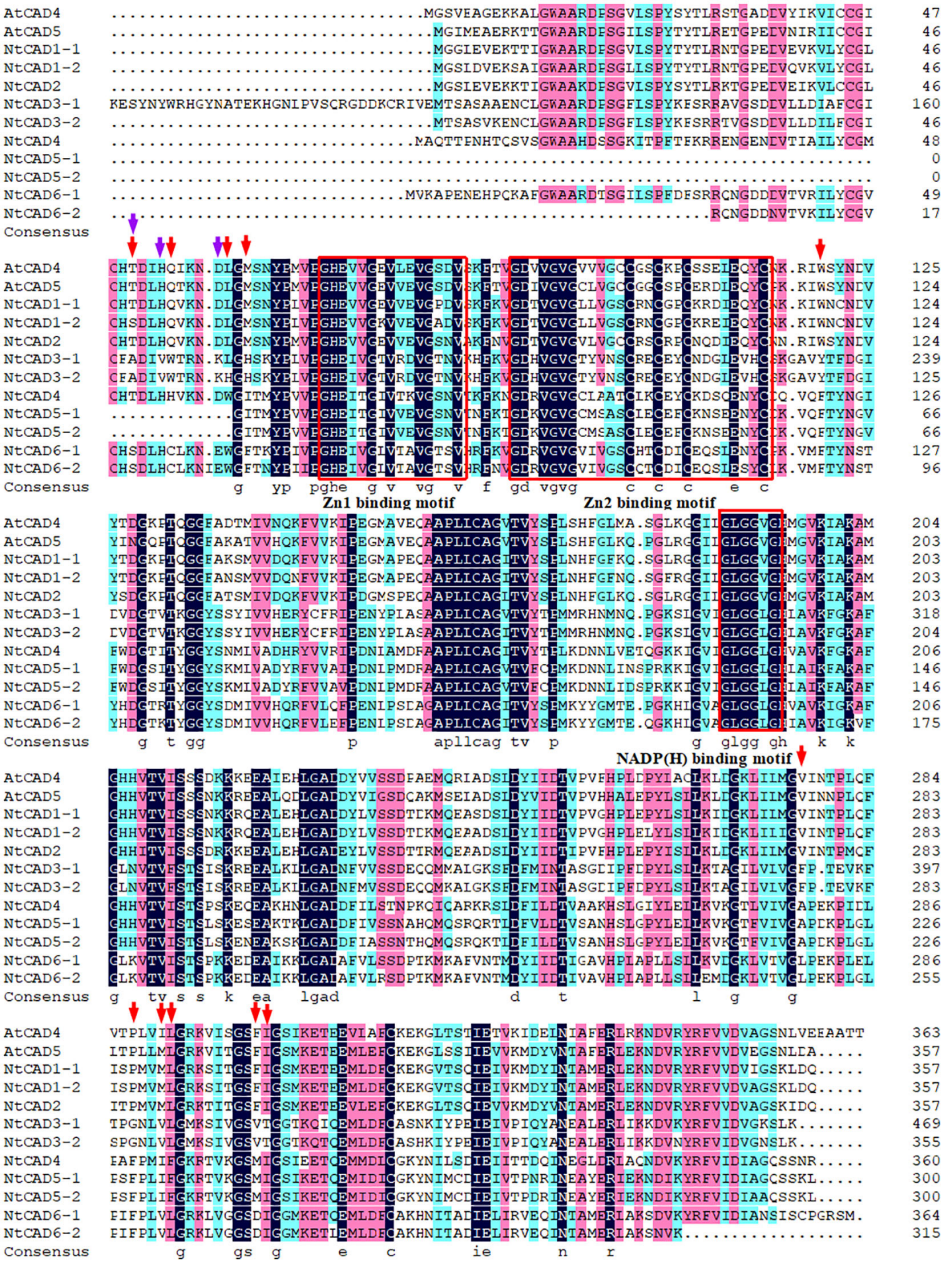
Alignment of NtCADs and *bona fide* Arabidopsis CADs. The *bona fide* AtCADs AtCAD4 (At3g19450) and AtCAD5 (At4g34230) were used as reference sequences. Zn-binding motifs are highlighted in red boxes, and the NADP(H)-binding motif is also indicated. The red and purple arrows denote the presence of conserved amino acids.

Alignment analysis demonstrated that all NtCADs exhibited a high degree of amino acid sequence conservation compared with the *bona fide Arabidopsis* CADs (AtCAD4 and AtCAD5). They shared identical residues at the critical Zn1 catalytic center (C47, H69, and C163) and displayed characteristic motifs commonly found in CAD proteins (**Figure 2**). Consistent findings were obtained from the analysis of CAD sequences in the two diploid tobacco plants (**Supplementary Figure 1**). The conserved motifs identified in all CADs included a GHE(X)2G(X)5G(X)2V motif and a GD(X)10C(X)2C(X)2C(X)7C motif, which are involved in the binding of catalytic Zn^2+^ and structural Zn^2+^, respectively. In addition, the GLGGV(L)G motif plays a role in NADP(H) cofactor binding. We also observed that NtCAD1–1, NtCAD1–2, NtCAD2, and their homologous genes (Nsyl0449860, Ntom0265640, and Nsyl0290350) exhibit a high degree of conservation in 11 proposed amino acids (T49, Q53, L58, M60, W119, V276, P286, M289, L290, F299, and I300) within the substrate-binding sites of *bona fide* CADs (Kim et al., 2004). In addition, we also found that NtCAD1–1, NtCAD1–2, and NtCAD2 all have three conserved amino acids (Thr49, His52, and Asp57) that play an important role in the reduction process of AtCAD5 (Youn et al., 2006). Conversely, other CADs displayed varying amino acid substitutions at the proposed residues (**Figure 2**; **Supplementary Figure 1**).

**Figure 3:**
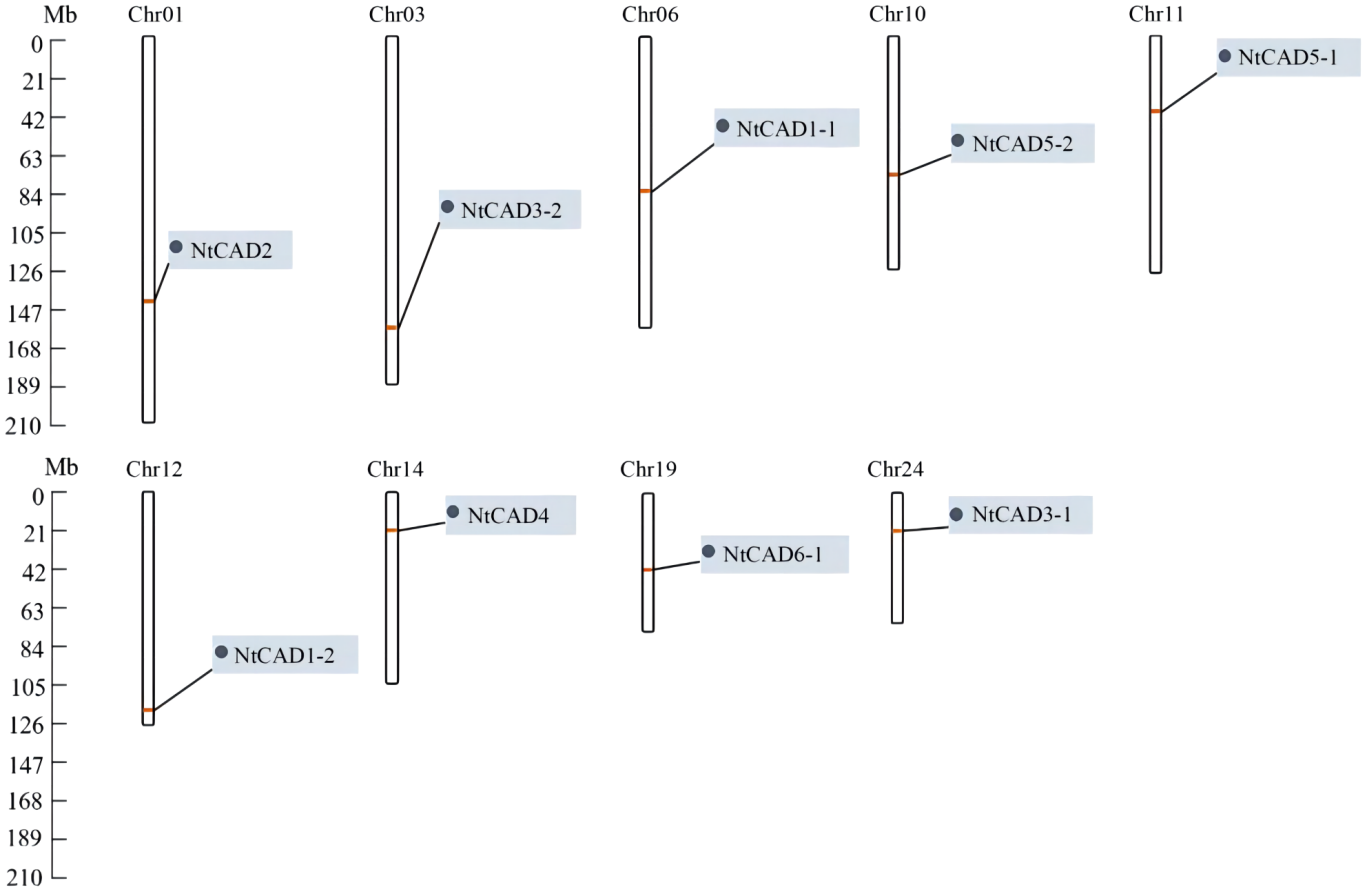
Localization of *CAD* gene family members on chromosomes in *Nicotiana tabacum*.

To determine the distribution of *CAD* genes on chromosomes, we retrieved physical maps of *CAD* genes from the China Tobacco Genome Database v3.0. Subsequently, we created simplified maps including information on chromosome length, start/end sites, and the numbers of *CAD* genes. As shown in **Figure 3**, chromosomes 1, 3, 6, 10, 11, 12, 14, 19, and 24 each harbored one *NtCAD* gene; additionally, one *NtCAD* gene was found to be located on a scaffold. Furthermore, in *N. sylvestris*, there are six *NsylCAD* genes, which were located on chromosomes 1, 2, 6, 7, 8, and 9, respectively (**Supplementary Figure 2A**), whereas four *NtomCAD* genes were located on chromosomes 5, 7, 10, and 12 (**Supplementary Figure 2B**) in *N. tomentosiformis*.

### 3.3 Intron-exon structure of tobacco *CAD* genes

**Figure 4:**
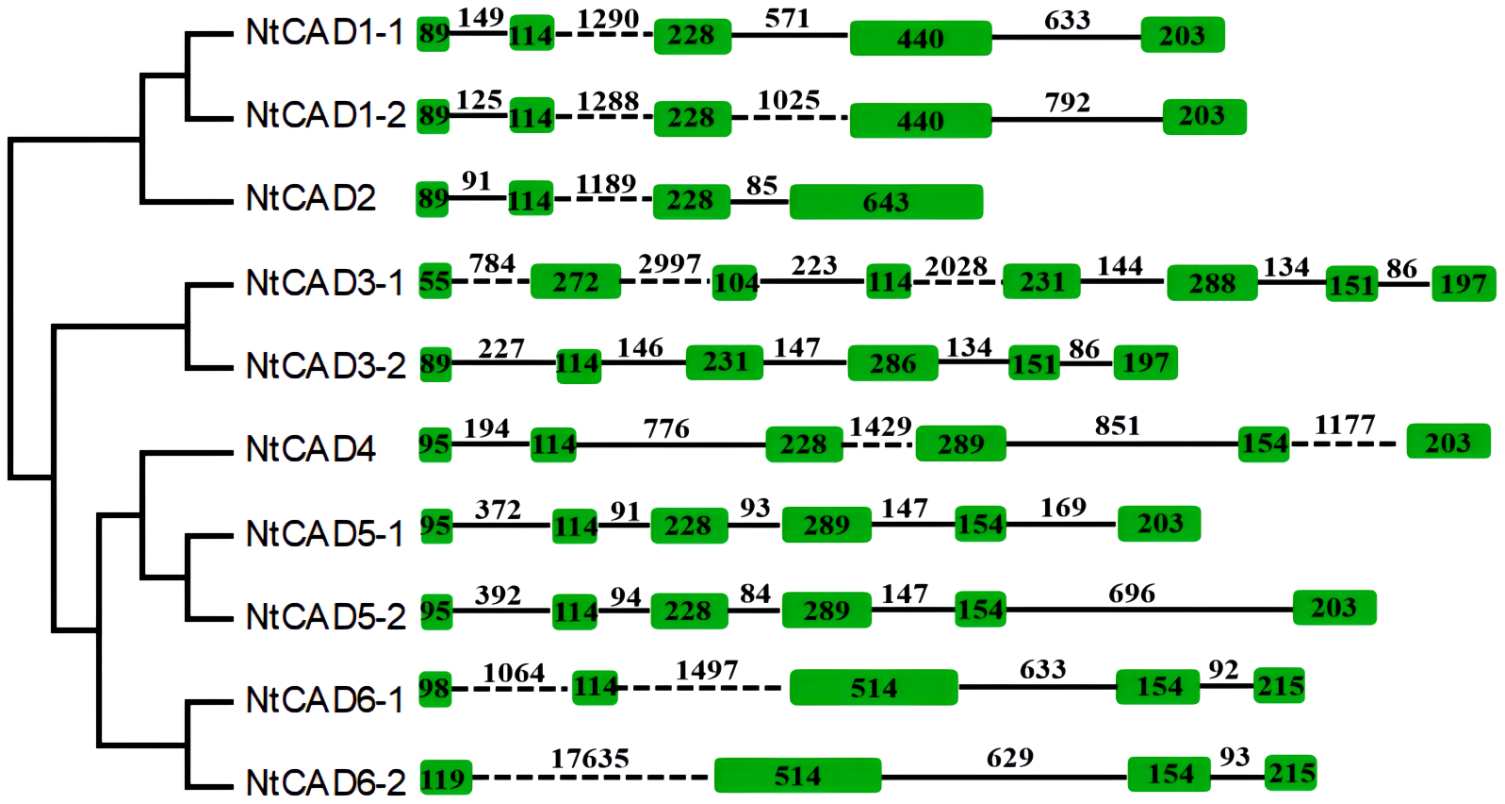
Analysis of the exon/intron structural organization of the 10 *NtCAD* genes. Introns and exons are indicated by solid lines and open boxes, respectively.

To explore the origins of the identified tobacco *CAD* genes, the exon/intron structure of each gene was analyzed. As depicted in **Figure 4**, there was significant variation in intron length among NtCADs. Most CAD introns ranged from 80 bp to 2,000 bp, with exceptions in *NtCAD3–1* and *NtCAD6–2*, which had introns exceeding 2,000 bp. Similarly, diploid tobacco exhibited variations in intron length. *Nsyl0426890* and *Nsyl0459450* both have one intron exceeding 3,000 bp (**Supplementary Figure 3**). Moreover, the number of introns varied among the 10 *NtCADs*. For example, there are four introns in *NtCAD1–1*, *NtCAD1–2*, and *NtCAD6–1*, respectively, whereas only three introns were observed in *NtCAD2* and *NtCAD6–2*. In addition, *NtCAD3–2*, *NtCAD4*, and *NtCAD5* had five introns each, and *NtCAD3–1* had seven introns. Most *CAD* genes in diploid tobacco genomes exhibited a consistent number of introns with their corresponding orthologs in *N. tabacum*; however, exceptions were noted for Nsyl0426890, Nsyl0054440, and their corresponding homologs, Ntom011853*0* and Ntom0224390 (**Figure 4**; **Supplementary Figure 3**).

### 3.4 Phylogenetic analysis of NtCAD proteins

To better assess the evolutionary relationships among CAD proteins in tobacco and other plant species, 65 full-length CAD amino acid sequences from 13 distinct species were analyzed. The phylogenetic classification revealed that CAD proteins could be grouped into three subfamilies (**Figure 1**). Group I comprises CAD1 and CAD2, along with *bona fide* CAD proteins like AtCAD4, AtCAD5 (Kim et al., 2004). NtCAD3 was classified into group IIa, along with AtCAD1, OsCAD1, and OsCAD4, which have been reported to exhibit no detectable CAD activity (Kim et al., 2004; Park et al., 2018). Group IIb comprises NtCAD4, NtCAD5, and OsCAD6, which was demonstrated to have CAD activity toward hydroxycinnamaldehydes (Park et al., 2018). Additionally, CAD6, classified under group III, is associated with AtCAD2, AtCAD3, and AtCAD9, which participate in plant defense mechanisms or function as functionally redundant *CAD* genes (Kim et al., 2004). Consistent with previous findings, highly similar CAD protein sequences clustered together within the same subgroup.

### 3.5 Expression analysis of tobacco *CAD* genes

**Figure 5:**
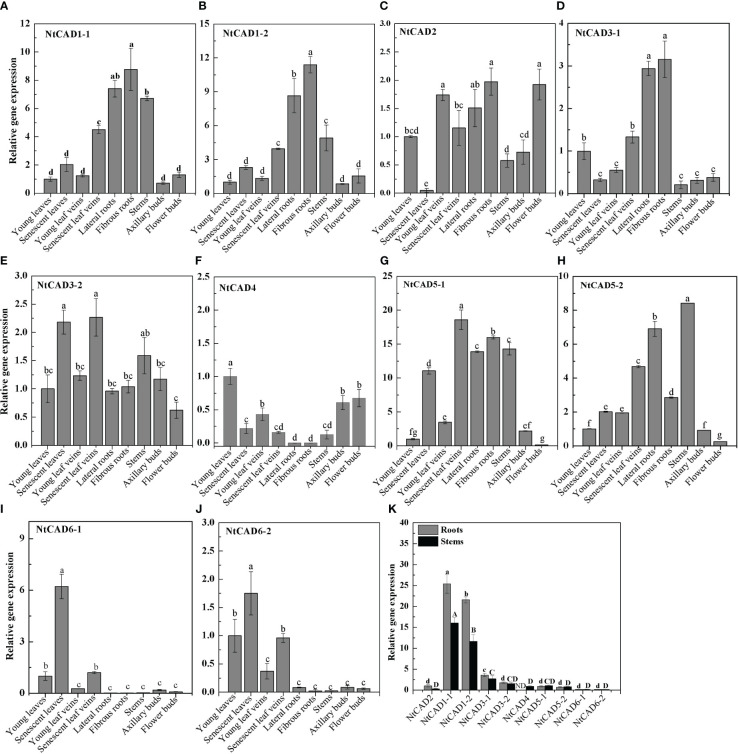
Expression profiles of *NtCAD* genes in different tobacco tissues at the flowering stage. Different tobacco tissues including young and senescent leaves, young and senescent leaf veins, lateral and fibrous roots, stems, and axillary and flower buds were samples from 4-month-old tobacco with half of the blooming flowers. Afterward, the expression levels of *NtCAD* genes **(A–J)** were detected by qRT-PCR. In addition, relative expression levels of *NtCAD* genes in roots and stems **(K)** were at the flowering stage. The tobacco *L25* gene was used as the internal control. Bars labeled with different letters indicate significant differences at *P* < 0.01 based on Duncan’s multiple test.

The physiological functions of genes can often be partially understood by analyzing their expression patterns. Therefore, qRT-PCR analysis was conducted to examine the expression levels of *NtCAD* genes in different tobacco tissues. As shown in **Figure 5**, *NtCAD1–1* and *NtCAD1–2* exhibit similar expression patterns, with higher expression levels were observed in lignified tissues such as roots and stems (**Figures 5A, B**). *NtCAD2* is mainly expressed in young leaf veins, fibrous roots, and flower buds (**Figure 5C**), suggesting probable tissue-specific functions for *NtCAD1* and *NtCAD2*. However, other *NtCAD* genes showed different expression preferences; for example, *NtCAD3–1* is preferentially expressed in roots (**Figure 5D**), *NtCAD3–2*, *NtCAD4*, *NtCAD6–1*, and *NtCAD6–2* in leaves (**Figures 5F, I, J**), as well as *NtCAD5–2* in stems (**Figure 5H**). In addition, the fold change of *NtCAD5* gene expression exceeds or is similar to that of *NtCAD1* gene expression in both leaves and roots. However, *NtCAD1–1* and *NtCAD1–2*, which belong to the *bona fide CAD* phylogenetic group, had the highest and second-highest expression levels in the roots and stems, respectively (**Figure 5K**). These two genes were expressed 11 to 25 times higher than *NtCAD2*, showing the mRNA transcript abundance of them was richer in such tissues, and implying that *NtCAD1–1* and *NtCAD1–2* are likely playing important roles in tobacco lignification.

### 3.6 Biochemical characterization of the recombinant NtCAD1 and NtCAD2 enzymes

Recombinant NtCAD1–1, NtCAD1–2, and NtCAD2 proteins were then successfully purified at concentrations ranging from 0.3 mg/mL to 0.7 mg/mL, with calculated theoretical molecular weights of 40.33 kDa, 40.22 kDa, and 40.42 kDa respectively. The successful expression of the fusion protein comprising NtCADs was confirmed by SDS-PAGE and western blot analyses (**Supplementary Figures 4**, **5**). These results suggest that the purified NtCADs exhibited slight deviations from their predicted molecular weights (**Table 1**), potentially due to factors such as fusion expression with the His-tag, posttranslational modifications, or experimental variations.

**Figure 6:**
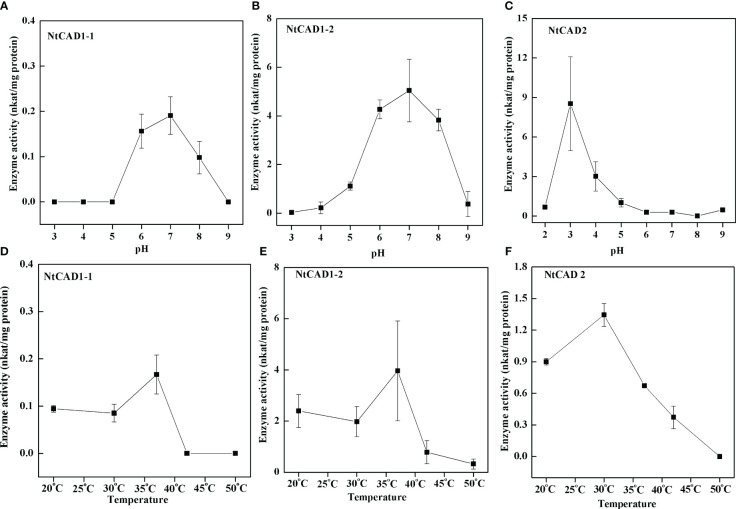
Optimal pH and temperature conditions for NtCAD1 and NtCAD2 utilizing coniferyl aldehyde as a substrate. **(A–C)**, pH profiles of NtCAD1–1 **(A)**, NtCAD1–2 **(B)**, and NtCAD2 **(C)** activities; **(D–F)**, Temperature profiles of NtCAD1–1 **(D)**, NtCAD1–2 **(E)**, and NtCAD2 **(F)**.

To assess the pH dependence and temperature sensitivity of the reduction activity of NtCAD1–1, NtCAD1–2, and NtCAD2, we conducted *in vitro* experiments using coniferyl aldehyde as the substrate (**Figures 6A–C**). The optimal pH values for the reduction activity were determined to be 7.0, 7.0, and 3.0 for NtCAD1–1, NtCAD1–2, and NtCAD2, respectively. Furthermore, the optimum temperatures were found to be 37°C, 37°C, and 30°C for NtCAD1–1, NtCAD1–2, and NtCAD2, respectively (**Figures 6D–F**).

The kinetic parameters of the reactions catalyzed by recombinant NtCAD1–1, NtCAD1–2, and NtCAD2 were determined using three distinct substrates: coniferyl aldehyde, *p*-coumaryl aldehyde, and sinapyl aldehyde (**Table 4**). All three enzymes exhibited NADPH-dependent reductase activity toward the assessed substrates. The *K_m_* values of the detected substrates indicated a closer proximity in value between NtCAD1–1 and NtCAD1–2 (2.95 µM–4.17 µM for NtCAD1–1 and 0.71 µM–2.00 µM for NtCAD1–2, **Table 4**). However, the *K_m_* values of NtCAD2 were significantly higher than those of NtCAD1–1 and NtCAD1–2, particularly for the sinapyl aldehydes (**Table 4**). In contrast, lower *V_max_* values were observed for NtCAD1–1 than for NtCAD1–2 or NtCAD2 (**Table 4**). As a result, the catalytic efficiencies (*K_cat_*/*K_m_* values) of NtCAD1–2 were found to be more than 6 to 178 times higher than those of NtCAD1–1 and NtCAD2, suggesting that NtCAD1–2 exhibits significantly higher catalytic activity than NtCAD1–1 or NtCAD2.

**Table 4:** Kinetic analysis of NtCAD1 and NtCAD2.

Protein symbol	Substrate	*K*_m_ (μM)	*V*_max_ (nkat mg^−1^ protein)	*K*_cat_ (s^−1^)	*K*_cat_/*K*_m_ (μM^−1^ s^−1^)
NtCAD1–1	Coniferyl aldehyde	2.95	0.16	0.007	0.002
	*p*-Coumaryl aldehyde	4.17	0.11	0.004	0.001
	Sinapyl aldehyde	4.01	0.15	0.006	0.001
NtCAD1–2	Coniferyl aldehyde	1.00	5.83	0.23	0.230
	*p*-Coumaryl aldehyde	2.00	6.79	0.27	0.135
	Sinapyl aldehyde	0.71	4.76	0.19	0.268
NtCAD2	Coniferyl aldehyde	4.97	2.48	0.10	0.020
	*p*-Coumaryl aldehyde	7.95	4.17	0.17	0.021
	Sinapyl aldehyde	18.75	3.74	0.15	0.008

### 3.7 Predicted protein structures of NtCAD1 and NtCAD2

**Figure 7:**
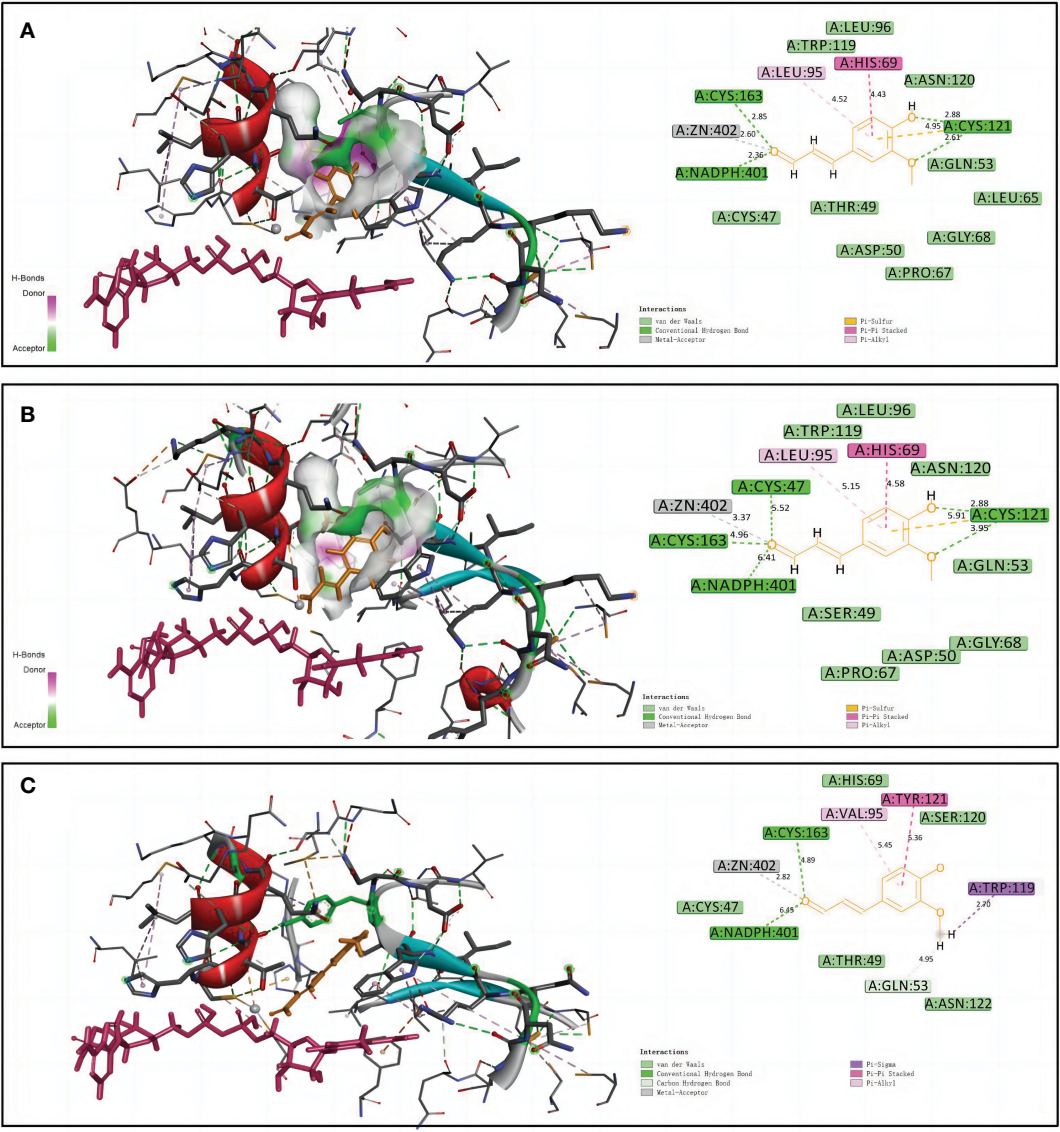
Structural modeling and docking analysis of NtCAD1–1, NtCAD1–2, and NtCAD2. Docking analysis was performed for NtCAD1–1 **(A)**, NtCAD1–2 **(B)**, and NtCAD2 **(C)** with coniferyl aldehyde as a ligand. Coniferyl aldehyde is depicted in orange, NADPH is represented in red, and the Zn atoms are illustrated in gray.

Homology models of NtCAD1–1, NtCAD1–2, and NtCAD2 were constructed and evaluated. The modeled structures of NtCAD1–1, NtCAD1–2, and NtCAD2 did not change significantly (**Supplementary Figure 6**) and clearly showed properly sized binding pockets for NADPH and the cinnamyl aldehyde substrate (**Figure 7**; **Supplementary Figure 6**). The size of the substrate-binding pocket in each NtCAD is similar, and the residues involved in interacting with coniferyl aldehydes were conserved. The amino acids at positions 69, 95, and 121 play a crucial role in assisting other amino acids to secure the substrate by interacting with its Pi bond in the benzene ring (**Figure 7**). However, the docking conformations of substrates with NtCAD1 and NtCAD2 were different. The Tyr-121 of the benzene side chain in NtCAD2 was replaced with Cys-121 in NtCAD1–1 and NtCAD1–2, resulting in a reduced limiting effect on the substrate and exposing the cavity, further expanding the reaction cavity. When the substrate binds to the protein, the benzene ring plane reverses outward, and the oxymethyl group on the benzene ring extends into the interior of the reaction cavity. This conformation may allow the substrate to bind more stably to the reaction cavity and provide broad spectrum conditions for the substrate’s oxymethyl side chain.

### 3.8 The effects of downregulation of *NtCAD1* and *NtCAD2* on lignin contents

**Figure 8:**
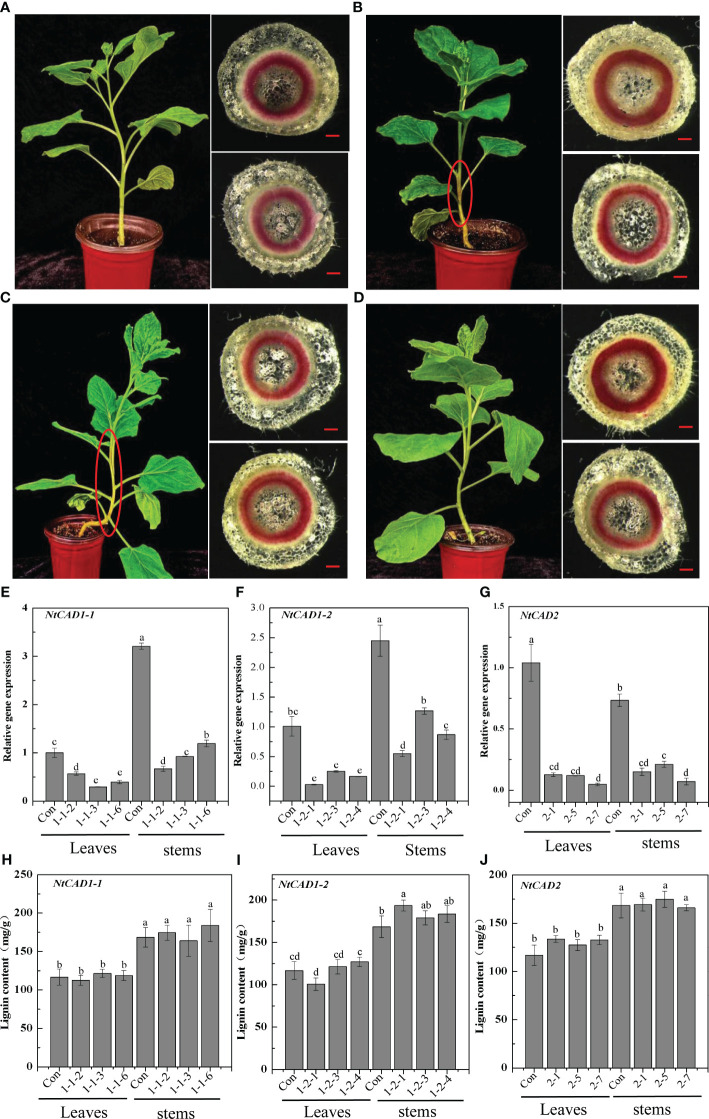
The downregulation of *NtCAD1–1*, *NtCAD1–2*, and *NtCAD2* does not exert any significant impact on the lignin content. **(A–D)**. Plant growth conditions and lignin phloroglucinol staining of transgenic tobacco with downregulated *NtCAD1–1*, *NtCAD1–2*, and *NtCAD2* using VIGS. Representative images of the Con (pTRV2, A), VIGS-NtCAD1–1 **(B)**, VIGS-NtCAD1–2 **(C)**, and VIGS-NtCAD2 **(D)** groups are shown. The scale bars indicate 40 μm; the red-brown stem is highlighted by a red circle in **(B, C)**; the expression levels of NtCAD1–1 **(E)**, NtCAD1-2 **(F)**, and NtCAD1–1 **(G)** in transgenic tobacco leaves and stems were assessed, whereas the lignin content of NtCAD1–1 **(H)**, NtCAD1-2 **(I)**, and NtCAD2 **(J)** was measured in both transgenic tobacco leaves and stems. Bars denoted by different letters indicate significant differences at *P* < 0.05, as determined by Duncan’s multiple-range test.

Downregulation of *NtCAD1* and *NtCAD2* was achieved by VIGS performance. Reddish-brown stems were observed in the VIGS-NtCAD1–1 and VIGS-NtCAD1–2 transgenic tobacco plants (**Figures 8B, C**). It is worth noting that only a small portion of the stems in VIGS-NtCAD1–1 transgenic tobacco plants exhibited red coloration in comparison with the VIGS-NtCAD1–2 transgenic tobacco. This observation aligns with the fact that NtCAD1–1 enzyme activity is lower than that of NtCAD1–2. The extent of downregulation of *NtCAD1–1*, *NtCAD1–2*, and *NtCAD2* in transgenic tobacco was quantified using qRT-PCR (**Figures 8E–G**). Significant downregulation of *NtCAD1–1* was observed in leaves and stems. Despite the significant decrease in *NtCAD1–1* expression (ranging from 43.07% to 70.55% in leaves and 62.74% to 79.14% in stems), there was no noticeable change in lignin content in these tissues (**Figure 8H**). Moreover, the downregulation of *NtCAD1–2* and *NtCAD2* in leaves and stems did not significantly affect lignin content (**Figures 8I, J**). Phloroglucinol staining was used to assess the lignin content in the secondary xylem of stems, also revealing no significant decrease (**Figures 8A–D**). Subsequently, the contents of *p*-hydroxyphenyl (H), guaiacyl (G), and syringyl (S) lignin-derived monomers in *NtCADs*-silenced plants were also detected by using GC-MS. As shown in **Table 5**, the lignin consists of a high content of the S unit when compared with the levels of the H and G units in whether control or *NtCAD*-silenced plants. In addition, the contents of both G and S monomers were significantly decreased when the *NtCAD* genes were silenced. However, the S/G ratio only decreased in *NtCAD1*-silenced plants.

**Table 5:** Gas chromatography-mass spectrometry identification and levels of the lignin monomers accumulated in stems of *NtCAD1* and *NtCAD2*-deficient tobaccos. Data are means ± SD of at least four independent samples.

	H+G+S (mg/g DW)	G (mg/g DW)	H (mg/g DW)	S (mg/g DW)	S/G ratio
Con	80.53 ± 1.93	16.61 ± 0.32	0.09 ± 0.00	63.83 ± 1.65	3.84 ± 0.05
NtCAD1–1-1	61.28 ± 2.15 **	15.69 ± 0.45 **	0.06 ± 0.01	45.52 ± 0.88 **	2.90 ± 0.03 **
NtCAD1–1-2	65.46 ± 1.85 **	16.01 ± 0.23 **	0.06 ± 0.01	49.38 ± 1.56 **	3.08 ± 0.07 **
NtCAD1–1-3	62.45 ± 2.47 **	15.54 ± 0.21 **	0.06 ± 0.00	46.84 ± 1.32 **	3.01 ± 0.01 **
NtCAD1–2-1	62.45 ± 1.18 **	13.97 ± 0.27 **	0.05 ± 0.00	48.43 ± 1.96 **	3.46 ± 0.14 *
NtCAD1–2-2	62.25 ± 1.45 **	13.88 ± 0.32 **	0.05 ± 0.01	48.31 ± 2.01 **	3.47 ± 0.11 **
NtCAD1–2-3	60.31 ± 2.01 **	13.41 ± 0.47 **	0.06 ± 0.00	46.84 ± 2.00 **	3.49 ± 0.05 **
NtCAD2–1	70.16 ± 3.21 **	14.84 ± 0.28 **	0.06 ± 0.00	55.27 ± 1.47 **	3.72 ± 0.14
NtCAD2–2	64.28 ± 1.25 **	13.50 ± 0.35 **	0.06 ± 0.00	50.71 ± 1.14 **	3.75 ± 0.12
NtCAD2–3	69.03 ± 2.14 **	14.29 ± 0.24 **	0.06 ± 0.01	54.67 ± 1.05 **	3.82 ± 0.09

Asterisks within columns are significantly different in comparison with corresponding Con samples at *P* < 0.05 and *P* < 0.01 (*t* test).

### 3.9 Diverse roles of *NtCAD1* and *NtCAD2* in response to abiotic and biotic stresses

Previous studies have indicated that *CAD* homologs play diverse roles in various plant biological processes, including their involvement in stress responses (Chao et al., 2022). To further investigate the potential role of *NtCAD1–1*, *NtCAD1–2*, and *NtCAD2* involved in plant regulatory networks, the 1,500-bp-length promoter regions upstream of the start codon (ATG) of each gene was analyzed. Subsequently, we analyzed these promoter sequences for cis-regulatory elements using the PlantCARE database (Lescot et al., 2002) and identified several cis-elements associated with plant growth and development (e.g., light responsiveness), biotic and abiotic stresses (e.g., cold stress, anaerobic induction), and phytohormone responsiveness including ABA, MeJA, and SA (**Supplementary Table 4**). In addition to conserved *cis*-elements shared with *NtCAD1–1* and *NtCAD1–2*, *NtCAD2* also possesses distinctive motifs, such as TC-rich repeats, which have been implicated in defense mechanisms and stress response (Zhang et al., 2021).

**Figure 9:**
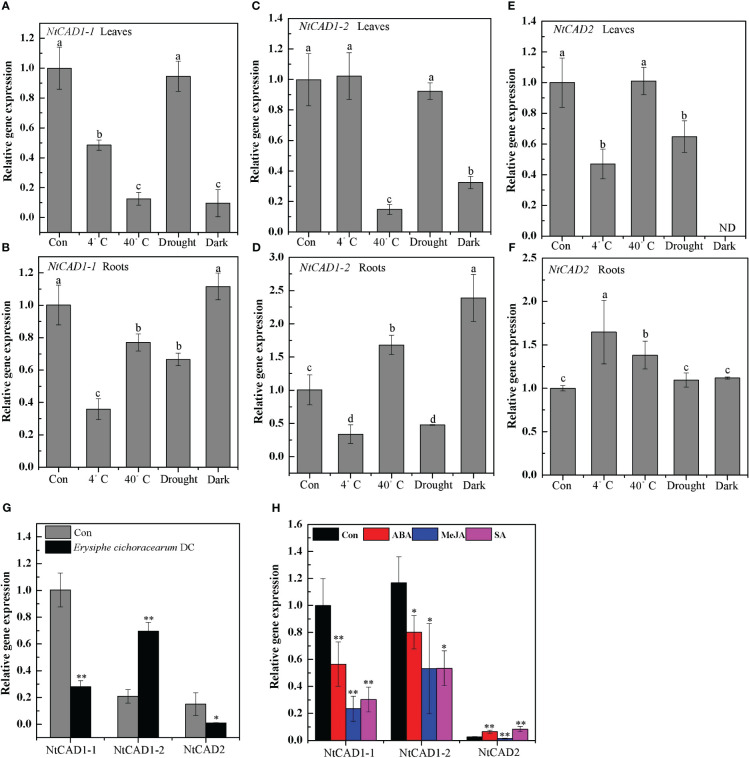
The expression profiles of NtCAD1–1, NtCAD1–2, and NtCAD2 in response to cold (4°C), heat (40°C), drought, dark stress **(A–F)**, fungus infection **(G)**, and ABA, MeJA, and SA treatments. The expression levels of the *NtCAD* genes were related to the internal control *L25* gene. The data represent means ± SD of at least four independent measurements obtained from different experiments. Significant differences between groups were determined using Duncan’s multiple test, with bars labeled with different letters **(A–F)** indicating significance at *P* < 0.05. Additionally, bars marked with asterisks **(G, H)** demonstrate significant differences compared to the control (Con) samples based on Student’s *t*-test (*P* < 0.05, *P* < 0.01).

The expression levels of *NtCAD* genes in response to abiotic and biotic stresses were subsequently determined. Notably, *NtCAD1–1* exhibited significant downregulation or minor effects in response to all tested biotic and abiotic stresses (**Figures 9A, B, G**). Distinct response patterns were observed for *NtCAD1–2* and *NtCAD2* in the leaves and roots (**Figures 9C–F**). Specifically, under heat and dark stress conditions, the expression of *NtCAD1–2* was significantly induced in roots but decreased in leaves (**Figures 9C, D**). Conversely, when exposed to cold or heat stress conditions, the expression of *NtCAD2* was significantly increased only in the roots; however, it displayed either significant downregulation or minor effects in leaves under the same stress conditions. With *Erysiphe cichoracearum* DC infection, diseased leaves exhibited a significant increment in *NtCAD1–2* expression compared with healthy leaves, whereas *NtCAD1–1* and *NtCDAD2* exhibited notable decreases (**Figure 9G**). Meanwhile, the *NtCAD*-silenced transgenic tobacco plants (VIGS-NtCAD1–1, VIGS-NtCAD1–2, and VIGS-NtCAD2) were then inoculated with *E. cichoracearum* DC. for 12 days. As expected, *E. cichoracearum* DC mycelium and spores were observed in all the inoculated plants. However, the symptoms were more severe in the *NtCAD*-silenced (especially for *NtCAD1–2*-silenced) transgenic tobacco than in the control plants. Similar results were also observed in withered leaves when inoculated with *E. cichoracearum* DC (**Supplementary Figure 7**).

Further investigations were conducted to analyze the expression patterns of these genes following ABA, MeJA, and SA treatment, considering the presence of specific motifs within their promoter regions known to be responsive to these treatments (**Supplementary Table 4**). Both *NtCAD1–1* and *NtCAD1–2* were significantly downregulated under ABA, MeJA, and SA treatments. Interestingly, the expression of *NtCAD2* was upregulated under both ABA and SA treatments (**Figure 9H**). These findings highlight the functional divergence of these three *NtCADs* in response to biotic and abiotic stresses in tobacco.

## 4 Discussion

The final step of the monolignol biosynthesis pathway is critically influenced by CAD, which is primarily responsible for lignin deposition and the formation of lignin components in secondary cell walls (Özparpucu et al., 2018). Comparative genomic analysis has been used to investigate multiple *CAD* gene families across diverse plant species, including *Arabidopsis*, rice, poplar, and oil palm. In this study, we identified 10 *CAD* genes in *N. tabacum*, 4 in *N. tomentosiformis*, and 6 in *N. sylvestris*. These tobacco CAD members exhibited significant similarities in their nucleotide and amino acid sequences, leading us to name them CAD1-CAD6 based on their unique molecular signatures. We further analyzed the genetic structure, evolutionary relationships, and expression patterns of NtCADs in different tobacco tissues. Additionally, enzyme activity assays and structural modeling were performed to characterize the putative *bona fide* NtCADs. Furthermore, we investigated the expression patterns of these three *bona fide* NtCADs under various biological and abiotic stresses, as well as in response to phytohormones. Therefore, this comprehensive functional analysis provides insights into the potential roles of *bona fide* NtCADs in various growth and developmental processes in tobacco.

### 4.1 Evolutionary conservation and divergence among tobacco *CAD* genes

By conducting a comprehensive analysis of the *CAD* gene family in tobacco, including examining conserved motifs, exon/intron gene structures, and phylogeny, researchers can derive generalizations and predictions regarding genetic and evolutionary relationships among uncharacterized members of this gene family. Moreover, this analysis facilitates the prediction of their potential functions. In this study, we conducted a phylogenetic analysis of CAD homologs from three tobacco species and compared them with those from 13 other plant species. Consistent with previous investigations (Barakat et al., 2009), our analysis classified tobacco CADs into three distinct clusters (**Figure 1**). CAD group I comprises *bona fide* CADs involved in lignin biosynthesis (Guo et al., 2010; Hirano et al., 2012; Liu et al., 2021), encompassing sequences from gymnosperm clades, monocots, and eudicots. Groups II and III contain sequences from both monocots and eudicots. This suggests that the development of group II and group III occurred in the precursor of angiosperms, or at least before the divergence of monocots and dicots. Our findings are consistent with those of previous analyses that classified the *CAD* genes in *Populus* into three classes, with gymnosperm sequences clustering in group I alongside monocots and eudicots (Barakat et al., 2009). However, the phylogenetic tree obtained in this study differs from a previously published tree, which classifies CADs into two or five main groups (Tobias and Chow, 2005; Hu et al., 2022), as well as from a previously published *Arabidopsis* tree, which demonstrates a distinct grouping of gymnosperm CAD sequences (Raes et al., 2003). This discrepancy observed between our phylogenetic tree and those previously published (Raes et al., 2003; Tobias and Chow, 2005; Hu et al., 2022) can be attributed to our incorporation of a more comprehensive range of species in this investigation.

Previous studies have demonstrated that green algae CADs typically possess seven to eight introns, whereas most land plants CADs exhibit fewer than six introns, such as *Populus*, watermelon, and pomegranate (Barakat et al., 2009; Guo et al., 2010; Jin et al., 2014; Hu et al., 2022). This finding is consistent with previous research indicating the complex structure of early eukaryotic genes (Roy and Gilbert, 2005). In this study, the structural analysis of isolated tobacco *CAD* genes revealed a varying number of introns, ranging from two to eight per gene (**Figure 4**; **Supplementary Figure 3**). However, most *CAD* genes in tobacco, like those in other land plants, typically possess four or five introns. The prevalence of low rates of intron gain and reduction in eukaryotic evolutionary processes (Roy and Gilbert, 2005; Roy and Penny, 2007) leads to the hypothesis that *CAD* genes with fewer introns are subjected to stronger selective pressure.

### 4.2 NtCAD1 is the main CAD involved in lignin biosynthesis in tobacco

Typically, one or two *bona fide* CADs are identified to fulfill essential functions in lignification (Kim et al., 2004; Tobias and Chow, 2005; Ma, 2010; Chao et al., 2014). Kim et al. (2004) reported that six AtCADs exhibit *in vitro* activity against hydroxycinnamaldehydes; however, only two *bona fide* AtCADs, AtCAD4 and AtCAD5, were identified as the predominant CADs involved in lignification. In *Populus*, PtrSAD, PtoCAD1, PtoCAD2, and PtoCAD8 demonstrate *in vitro* activity against hydroxycinnamaldehydes (Bomati and Noel, 2005; Chao et al., 2014); however, only PtoCAD1, which is classified within the *bona fide* clade, participates in the process of lignification. Similarly, in mulberry, MaCAD1, MaCAD2, MaCAD3/4, and MaCAD5 demonstrate activity against hydroxycinnamaldehydes *in vitro*. Among these, MaCAD3/4, classified as a *bona fide* member of the CAD family, is considered the primary CAD involved in lignification (Chao et al., 2014). In the present study, NtCAD1–1, NtCAD1–2, and NtCAD2, belonging to group I, emerged as the most promising *bona fide* CAD candidates in tobacco. NtCAD1–1 and NtCAD1–2 showed the highest and second-highest expression (11- to 25-fold higher than NtCAD2) among *NtCADs* in roots and stems, which accumulate lignin (**Figure 5**). Interestingly, similar expression patterns have been observed for other *bona fide* CADs, such as rice *OsCAD2*, which also demonstrates the highest expression levels in both roots and stems (Tobias and Chow, 2005). In contrast, wheat *bona fide TaCAD1* exhibits high expression exclusively in stem tissues (Ma, 2010). These findings suggest that *NtCAD1–1* and *NtCAD1–2* may play significant roles in lignification.

Enzymatic activity analysis confirmed the hypothesis that *NtCAD1* is the predominant *CAD* gene in tobacco. All three recombinant proteins exhibited significant *in vitro* activity against hydroxycinnamaldehydes (**Table 4**). Notably, NtCAD1–2 displayed catalytic activity ranging from 115 to 268 times higher than that of NtCAD1–1 (**Table 4**). These findings are consistent with those of previous studies in *Arabidopsis*, where AtCAD5 showed approximately 270 times higher catalytic activity than AtCAD4 (Kim et al., 2004). However, the catalytic efficiency of NtCAD1–2 was comparable with well-established CADs from other plant species, such as rice and mulberry (Hirano et al., 2012; Chao et al., 2022). The enzymatic conversion of coniferyl aldehyde to coniferyl alcohol using expressed and purified NtCAD1–1, 1–2, and 2 proteins showed significant pH dependence (**Figure 6**), with optimal activity observed at pH 7.0, 7.0, and 3.0 respectively. The difference in the optimal pH between BdCAD3 and BdCAD5 in *Brachypodium distachyon* can be partially attributed to the variation in the motif at positions 57–58 (Bukh et al., 2012). BdCAD3 possessed an EW motif, whereas BdCAD5 possessed an HL motif. The residue in AtCAD5 was identified as Asp, which is potentially crucial for the catalytic mechanism (Youn et al., 2006; Saathoff et al., 2011). CADs possessing the HL or DL motif and demonstrating notable catalytic activity toward monolignols are linked to cell wall lignification (Saathoff et al., 2011). However, the presence of a DL motif in all three NtCADs implies that additional residues may contribute to regulating optimum catalytic pH. Previous studies indicated that the diverse catalytic capabilities and functions of various CAD- and CAD-like enzymes in plants are not solely dictated by substrate-binding sites (Bukh et al., 2012). Similarly, we observed that despite the conservation of residues in the substrate-binding site of both NtCAD1–1 and NtCAD1–2, a single residue alteration has the potential to modulate enzymatic catalytic activity by inducing changes in the orientation of the substrate benzene rings (**Figure 7**).

Knocking down the *NtCAD1–1*, *NtCAD1–2*, and *NtCAD2* genes resulted in only a minimal or negligible reduction in lignin content (**Figures 8H–J**). Additionally, reddish-brown stems were observed in plants with knocked-down NtCAD1–1 or NtCAD1–2 (**Figures 8B, C**). This finding is consistent with previous research conducted on tobacco, where CAD downregulation plants exhibited a distinct red coloration of the xylem but showed only a slight decrease in lignin content (Stewart et al., 1997; Bernard Vailhé et al., 1998). Earlier studies provided evidence suggesting that the conversion of cinnamaldehyde to cinnamyl alcohols is not the rate-limiting step in lignin biosynthesis (Higuchi et al., 1994). Furthermore, disrupting the expression of AtCAD5 had minimal impact on overall lignin deposition (Kim et al., 2004). The same phenomenon was observed in alfalfa, wherein the downregulation of the CAD enzyme did not result in any significant alteration in lignin content (Baucher et al., 1999). This is primarily attributed to the compensatory action of alternative phenolic compounds and other CAD homologs (Guo et al., 2010). However, some studies have demonstrated that suppressing CAD leads to alterations in the lignin composition of transgenic plants while leaving the lignin content unaffected (Baucher et al., 1999; Fornalé et al., 2012; Trabucco et al., 2013). Therefore, the red stem phenotype generated by the knockdown of NtCAD1–1 or NtCAD1–2 (**Figures 8B, C**) may be attributed to the accumulation or enrichment of coniferyl aldehydes (Li et al., 2008b). Considering the high level of gene expression and enzyme activity of NtCAD1 coupled with concomitant phenotypic alterations, it is postulated that NtCAD1 may function as the main CADs in tobacco.

### 4.3 *Bona fide* NtCADs exhibit distinct expression patterns in response to abiotic and biotic stresses

Previous research indicates that certain *bona fide* CADs are not only involved in lignin biosynthesis but also actively participate in responding to both abiotic and biotic stresses. In flax, LuCAD1 and LuCAD2, classified as *bona fide* members of class I, have been found to respond to drought and cold stress, except for their role in lignification of maturing stems (Preisner et al., 2018). Similarly, the *bona fide* OsCAD2 in rice is induced by both biotic and abiotic stresses like pathogen infection and UV irradiation, implying that OsCAD2 contributes to developmental lignification and stress responses (Park et al., 2018). Our findings align with these studies, indicating that NtCAD1–2 is significantly upregulated in response to heat, dark stress, and pathogen infection. Furthermore, NtCAD2 demonstrates enhanced expression levels in roots under cold and heat stress conditions and in response to ABA and SA treatments, both pivotal in biotic and abiotic stress responses (Wu et al., 2019; Yu et al., 2019). However, we also observed a negative correlation between NtCAD1–1 expression and responses to abiotic and biotic stresses, as well as phytohormones. Similar to the mulberry findings reported by Chao et al. (2022), *bona fide* MaCAD3/4 expression was negatively associated with responses to abiotic and biotic stresses. These findings suggest that NtCAD1–2 functions as the main CAD, also participating in both abiotic and biotic stresses, whereas NtCAD2 may play a significant role in the response to various stresses.

## 5 Conclusion

In this study, 10 *CAD* genes were identified in *N. tabacum*, 4 in *N. tomentosiformis*, and 6 in *N. sylvestris*. Through genetic evolutionary analysis, we identified three putative *bona fide NtCAD* genes from *N. tabacum*. Gene expression assays conducted across different tobacco tissues provided compelling evidence supporting the potential involvement of NtCAD1–1 and NtCAD1–2 in lignin biosynthesis as they exhibited high expression levels in roots and stems. Enzyme activity analysis revealed that both NtCAD1 and NtCAD2 exhibited *in vitro* activity against hydroxycinnamaldehydes, with the highest efficiency observed for NtCAD1–2. Downregulation of NtCAD1–1 and NtCAD1–2 led to reddish-brown stems without significant altering lignin content; this observation can be attributed to the accumulation or enrichment of coniferyl aldehydes. Further investigations showed that these three NtCADs exhibit differential responses to biotic/abiotic stresses and phytohormones; notably, significant decreases or minor effects were observed for all tested conditions on the expression of NtCAD1–1 whereas heat stress, dark stress, and pathogen infection induced the expression of NtCAD1–2; conversely, cold/heat stress along with ABA and SA treatments induced the expression of NtCAD2. In summary, our findings provide a comprehensive analysis of NtCADs in tobacco and highlight the potential role of *NtCAD1* and *NtCAD2* in lignin biosynthesis. However, further investigation is required to elucidate the precise mechanism underlying the unaltered lignin content following NtCAD1 downregulation.

## Data availability statement

The datasets presented in this study can be found in online repositories. The names of the repository/repositories and accession number(s) can be found in the article/**Supplementary Material**.

## Author contributions

MW: Formal analysis, Investigation, Methodology, Validation, Writing – original draft, Writing – review & editing. YL: Investigation, Methodology, Validation, Writing – review & editing. ZTL: Investigation, Software, Writing – review & editing. LX: Investigation, Software, Writing – review & editing. YX: Investigation, Methodology, Validation, Writing – review & editing. LZ: Data curation, Investigation, Methodology, Validation, Writing – review & editing. XY: Data curation, Investigation, Methodology, Writing – review & editing. ZFL: Investigation, Software, Writing – review & editing. XX: Formal analysis, Investigation, Writing – review & editing. LW: Investigation, Methodology, Writing – review & editing. RW: Formal analysis, Investigation, Methodology, Writing – review & editing. SX: Writing – review & editing. JY: Writing – review & editing.
