# Supplementary material for: Genome-wide identification of the CAD gene family and functional analysis of putative bona fide CAD genes in tobacco (Nicotiana tabacum L.)
Source: Front Plant Sci. 2024 Jul 8;15:1400213. doi: 10.3389/fpls.2024.1400213 (PMC11261167; doi:10.3389/fpls.2024.1400213)
Supplement: Supplementary file 1 [file DataSheet_1.docx]

Supplementary Material

Genome-wide identification of *CAD* gene family and functional analysis of putative *bona fide* *CAD* genes in tobacco (*Nicotiana tabacum* L.)

Mingzhu Wu^1,#^, Yijun Li^2,#^, Zhengtai Liu^2,3^, Lin Xia^1^, Yiyu Xiang^2,3^, Lijie Zhao^1^, Xiaobei Yang^2^, Zefeng Li^1^, Xiaodong Xie^2^, Lin Wang^4^,Ren Wang^2,3^ Sheng Xu^2*^,Jun Yang^1*^

^1^China Tobacco Gene Research Center, Zhengzhou Tobacco Research Institute of CNTC, Zhengzhou, China

^2^Nanjing University of Chinese Medicine, Nanjing 210023, China

^3^Institute of Botany, Jiangsu Province and Chinese Academy of Sciences, Nanjing, 210014, P.R. China

^4^College of Life Science, Henan Agricultural University, Zhengzhou 450002, China

*** Correspondence:**Jun Yang
[yangjun@ztri.com.cn](mailto:yangjun@ztri.com.cn)

Sheng Xu

[xusheng@cnbg.net](mailto:xusheng@cnbg.net)

**Supplementary Table 1.** The PCR primer sequences for qRT-PCR.

| Accession number | Primer name | Sequences (5’→3’) |
| --- | --- | --- |
| Ntab0095940 | Ntab0095940-F | AGTATGTCACTCCGACTTG |
|  | Ntab0095940-R | ATCACTCCAACTCCAACTC |
| Ntab0420320 | Ntab0420320-F | TCACCTACAACTCCACATAC |
|  | Ntab0420320-R | TCCAATCTTCACAGCAACA |
| Ntab0152820 | Ntab0152820-F | ATCCATACTTGTCGCTGTTA |
|  | Ntab0152820-R | GCTCTTCATACCGAGAACTA |
| Ntab0868330 | Ntab0868330-F | GCCATATCCTAATCCACCAT |
|  | Ntab0868330-R | ATCTCCTCGTTGACTAACAG |
| Ntab0181830 | Ntab0181830-F | CCATTGCGATCTTGTATTGT |
|  | Ntab0181830-R | CCTACTCTATCTCCATTCTTGA |
| Ntab0336770 | Ntab0336770-F | GCAAGATCACACCTTACATT |
|  | Ntab0336770-R | CAGGAACAACAGGATACATAG |
| Ntab0554550 | Ntab0554550-F | GCTATACCAGACAACCTACC |
|  | Ntab0554550-R | TTCCTTGGCGAGTTAATCAA |
| Ntab0529780 | Ntab0529780-F | GGAGAAGTGGTGGAGGTA |
|  | Ntab0529780-R | CCATCAGTGTAGACATCATTG |
| Ntab0262430 | Ntab0262430-F | GATTACTCGTTGGAAGTTGTAG |
|  | Ntab0262430-R | CATCAGTGTAGACATCATTGC |
| Ntab0704510 | Ntab0704510-F | ATGAAGTGGTTGGTGAAGTT |
|  | Ntab0704510-R | TTGCCATCAGAGTAGACATC |
| L25 | L25-F | CCCCTCACCACAGAGTCTGC |
|  | L25-R | AAGGGTGTTGTTGTCCTCAATCTT |

**Supplementary Table 2.** The primer sequences for cloning the *CAD* gene and constructing the VIGS vector

| Accession number | Primer name | Sequences (5’→3’) |
| --- | --- | --- |
| *Ntab0529780* | NtCAD1-1-clone-F | ATGGTGGCTTGGAAGTTGAGAA |
|  | NtCAD1-1-clone-R | CATATCCTATTATTCGGTACGG |
| *Ntab0262430* | NtCAD1-2-clone-F | ATGGGTAGCTTGGATGTTGAA |
|  | NtCAD1-2-clone-R | GTAAGTTTGCATGGTTGGTA |
| *Ntab0704510* | NtCAD2-clone-F | ATGGGTAGTTTGGAAGTTGA |
|  | NtCAD2-clone-F | CCAAGAGCTTCTCACCTT |
| *Ntab0529780* | NtCAD1-1-VIGS-F | TCGACGACAAGACCCTGCAGGCCATCCTCTTGAACCTTAT |
|  | NtCAD1-1-VIGS-R | TGAGGAGAAGAGCCCTGCAGGCTTGCTTCCAATAACATCA |
| *Ntab0262430* | NtCAD1-2-VIGS-F | TCGACGACAAGACCCTGCAGAAGAAGCTGCTGATTCACT |
|  | NtCAD1-2-VIGS-R | TGAGGAGAAGAGCCCTGCAGTTCTCCAACCTCTCCATTG |
| *Ntab0704510* | NtCAD2-VIGS-F | TCGACGACAAGACCCTGCAGCAATGTCGCCAAGTTCAAT |
|  | NtCAD2-VIGS-F | TGAGGAGAAGAGCCCTGCAGTGCCTCCTCTTAATCCACT |

**Supplementary Table 3.** Identity matrix of predicted *CAD* genes and their coding amino acid sequences in *N. sylvestris* and *N. tomentosiformis*.

|  | | **Amino acid identity** | | | | | | | | | |
| --- | --- | --- | --- | --- | --- | --- | --- | --- | --- | --- | --- |
|  |  | **Nsyl0449860** | **Ntom0265640** | **Nsyl0290350** | **Nsyl0426890** | **Ntom0118530** | **Nsyl0093350** | **Ntom0311040** | **Nsyl0459450** | **Nsyl0054440** | **Ntom0224390** |
| **Nucleotide identity** | **Nsyl0449860** |  | 95.52 | 83.19 | 30.37 | 46.93 | 46.67 | 46.67 | 45.86 | 50.55 | 51.37 |
|  | **Ntom0265640** | 96.74 |  | 85.43 | 30.02 | 46.37 | 46.67 | 46.67 | 45.86 | 50.00 | 50.82 |
|  | **Nsyl0290350** | 83.61 | 84.64 |  | 28.06 | 43.58 | 44.44 | 44.44 | 42.82 | 47.25 | 48.08 |
|  | **Nsyl0426890** | 36.05 | 36.17 | 34.34 |  | 60.89 | 29.96 | 30.14 | 28.80 | 33.86 | 33.69 |
|  | **Ntom0118530** | 56.08 | 56.17 | 54.13 | 61.26 |  | 54.37 | 54.46 | 54.25 | 55.83 | 55.92 |
|  | **Nsyl0093350** | 55.49 | 55.40 | 54.80 | 36.00 | 46.54 |  | 96.94 | 75.41 | 54.79 | 55.62 |
|  | **Ntom0311040** | 56.05 | 55.59 | 55.63 | 36.00 | 47.09 | 96.31 |  | 75.41 | 54.52 | 55.34 |
|  | **Nsyl0459450** | 55.87 | 55.32 | 52.53 | 35.55 | 45.11 | 77.04 | 77.04 |  | 55.89 | 55.07 |
|  | **Nsyl0054440** | 55.29 | 54.61 | 54.84 | 36.30 | 45.15 | 59.02 | 58.93 | 61.02 |  | 97.53 |
|  | **Ntom0224390** | 56.52 | 55.62 | 54.89 | 36.03 | 44.37 | 59.20 | 59.02 | 61.29 | 97.81 |  |

**Supplementary Table 4.** Cis-regulatory elements present in the putative promoter regions of the *NtCAD1-1*, *NtCAD1-2*, and *NtCAD2* genes.

| **Gene cis-element** | **Annotation** | **Function** |
| --- | --- | --- |
| AAGAA-motif | Phytohormone responsive | cis-acting element involved in the abscisic acid responsiveness |
| ABRE | Phytohormone responsive | cis-acting element involved in the abscisic acid responsiveness |
| CGTCA-motif | Phytohormone responsive | cis-acting regulatory element involved in the MeJA-responsiveness |
| TGACG-motif | Phytohormone responsive | cis-acting regulatory element involved in the MeJA-responsiveness |
| TCA-element | Phytohormone responsive | cis-acting element involved in salicylic acid responsiveness |
| AE-box | Plant growth and development | part of a module for light response |
| Box 4 | Plant growth and development | part of a conserved DNA module involved in light responsiveness |
| G-box | Plant growth and development | cis-acting regulatory element involved in light responsiveness |
| GATA-motif | Plant growth and development | part of a light responsive element |
| GT1-motif | Plant growth and development | light responsive element |
| CAT-box | Plant growth and development | cis-acting regulatory element related to meristem expression |
| Sp1 | Plant growth and development | light responsive element |
| Gap-box | Plant growth and development | part of a light responsive element |
| W box | Biological and abiotic stresses | cold stress |
| ARE | Biological and abiotic stresses | cis-acting regulatory element essential for the anaerobic induction |
| GC-motif | Biological and abiotic stresses | enhancer-like element involved in anoxic specific inducibility |
| TC-rich repeats | Biological and abiotic stresses | cis-acting element involved in defense and stress responsiveness |

**Supplementary Figure 1.** Alignment of the protein sequences of NtomCADs, NsylCADs, and *bona fide Arabidopsis* CADs was performed using AtCAD4 (At3g19450) and AtCAD5 (At4g34230) as reference CADs. The red boxes indicate the presence of zinc binding motifs and the NADP(H) binding motif, while the red arrows denote the presence of conserved amino acids.

**Zn1 binding motif**

**Zn2 binding motif**

**NADP(H) binding motif**

**Supplementary Figure 2.** Chromosomal localization of *CAD* gene family members on the chromosomes of *N. sylvestris* (A) and *N. tomentosiformis* (B).


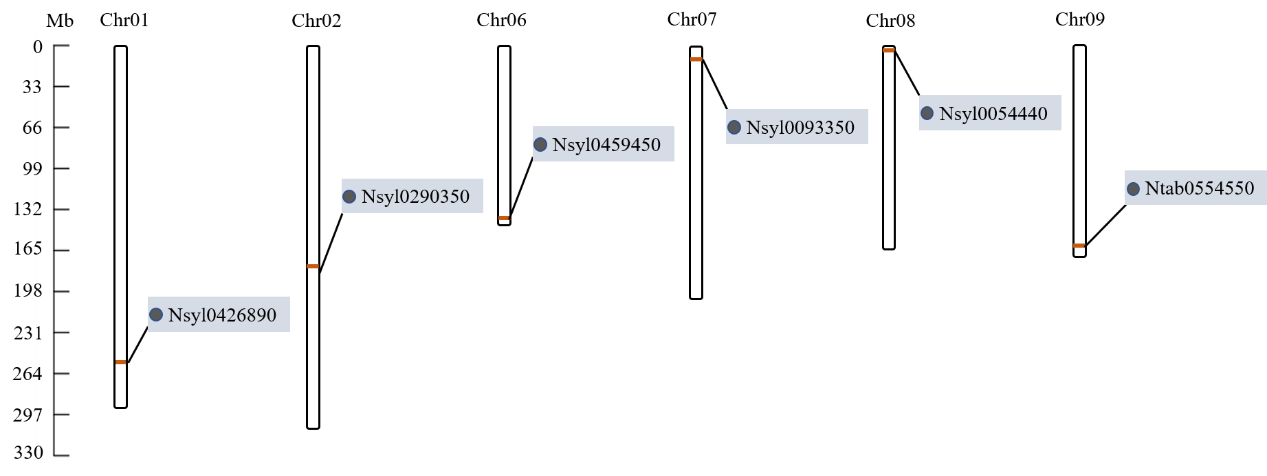

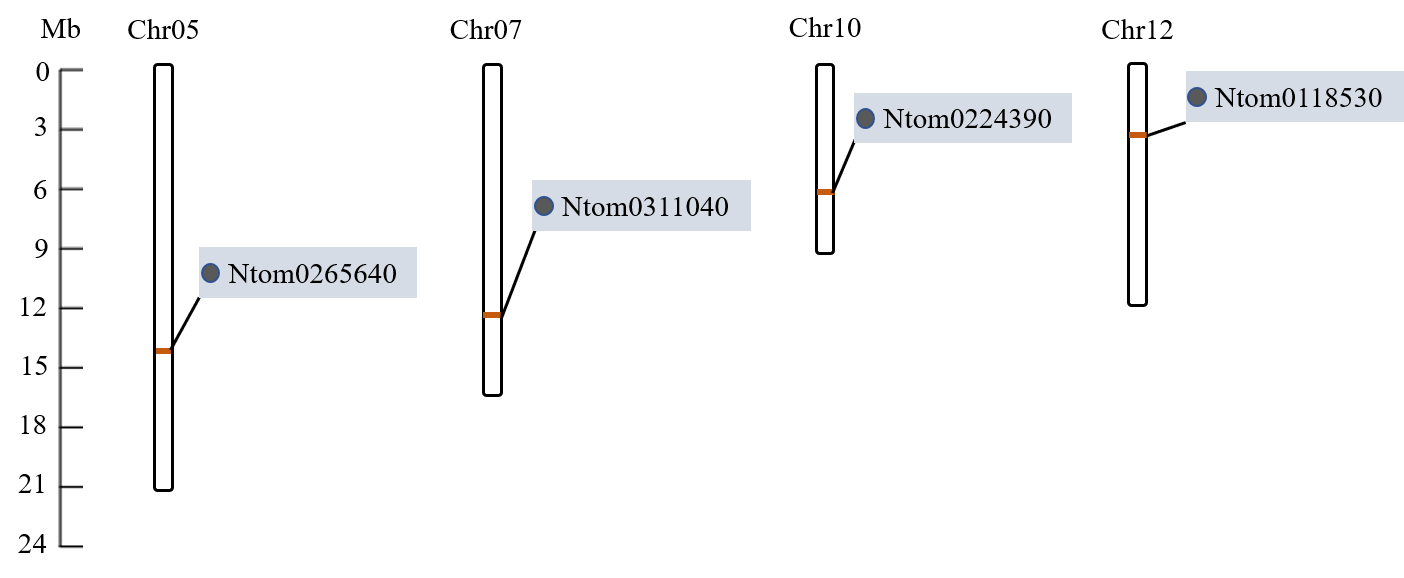


**A=**

**B=**


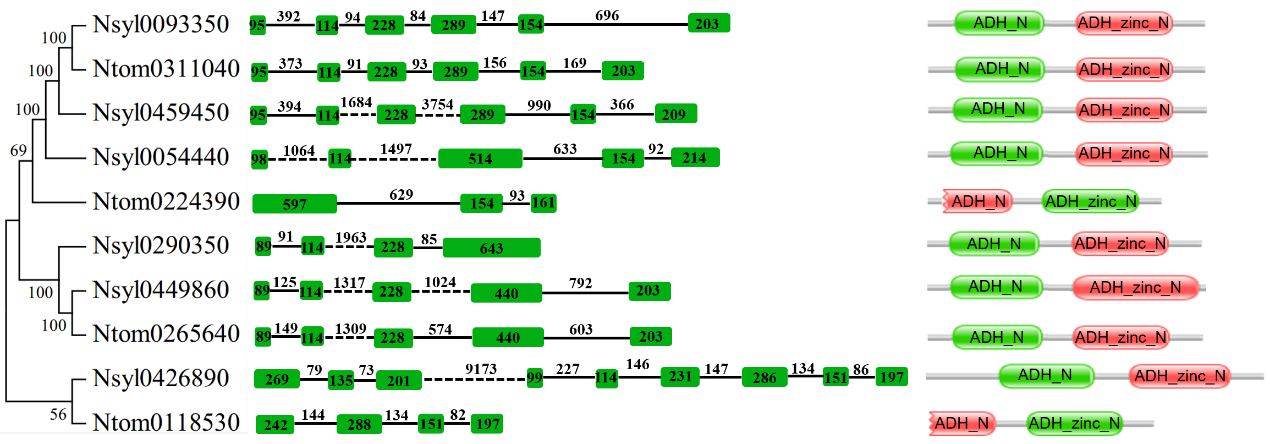
**Supplementary Figure 3.** Analysis of the exon/intron structural organization in the six *NsylCAD* and four *NtomCAD* genes.


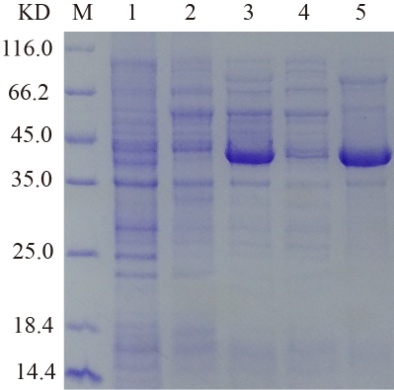

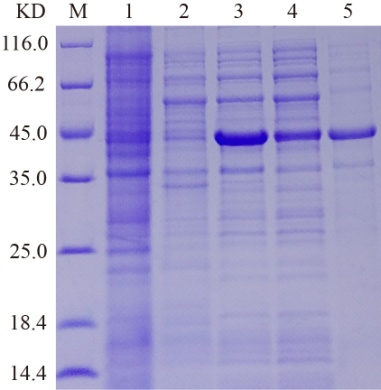

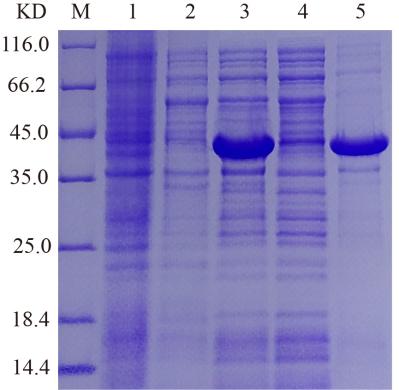


NAD1-1

NAD1-2

NAD 2

**Supplementary Figure 4.** The identification of protein expression for NAD1-1, NAD1-2, and NAD2 was conducted through SDS-PAGE analysis. M: Protein molecular weight marker; 1: Induced pET-28(a) empty vector; 2: Uninduced protein sample; 3: Induced protein sample; 4: Supernatant obtained after induced cell lysis; 5: Pellet obtained after induced cell lysis.


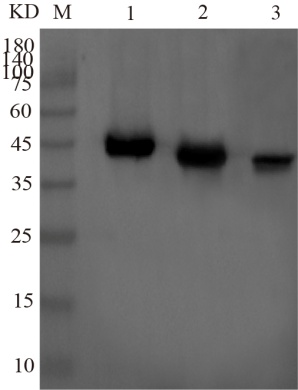
**Supplementary Figure 5.** The identification of protein expression for NAD1-1, NAD1-2, and NAD2 was conducted through Western blot analysis. M: Protein molecular weight marker; 1: NAD1-1 protein; 2: NAD1-2 protein; 3: NAD2 protein.


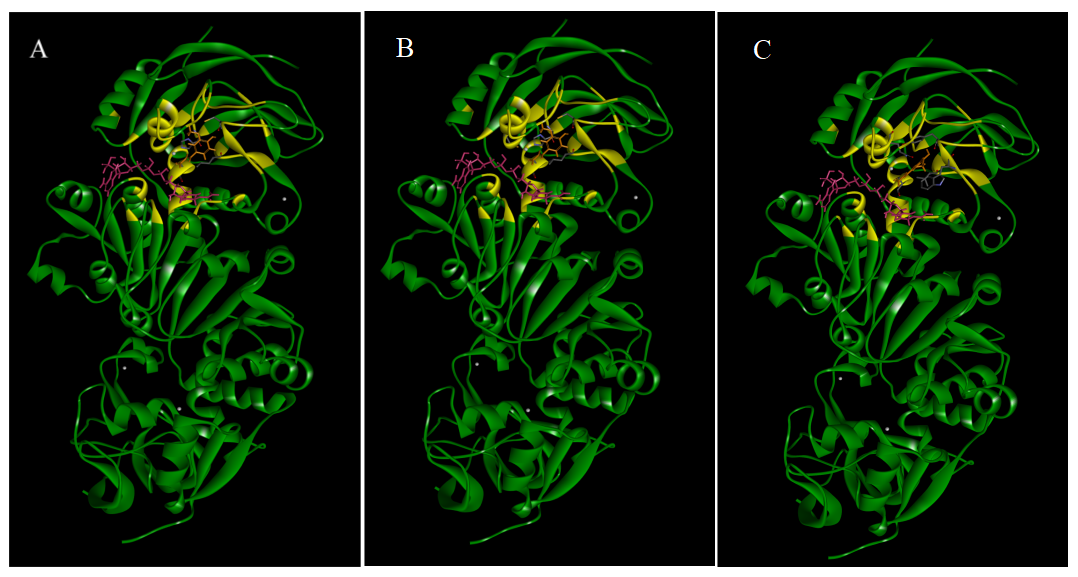


**Supplementary Figure 6.** A stereoview depicting the distribution of structural elements of NtCAD1-1 (A), NtCAD1-2 (B) and NtCAD2 (C). Coniferyl aldehyde is depicted in orange, NADPH is represented in red, the Zn atoms are illustrated in gray, and amino acids surrounding the substrate within 10 Å are shown in yellow.

**Supplementary Figure 7.** Disease development on transgenic tobaccos with *E. cichoracearum* DC inoculation.


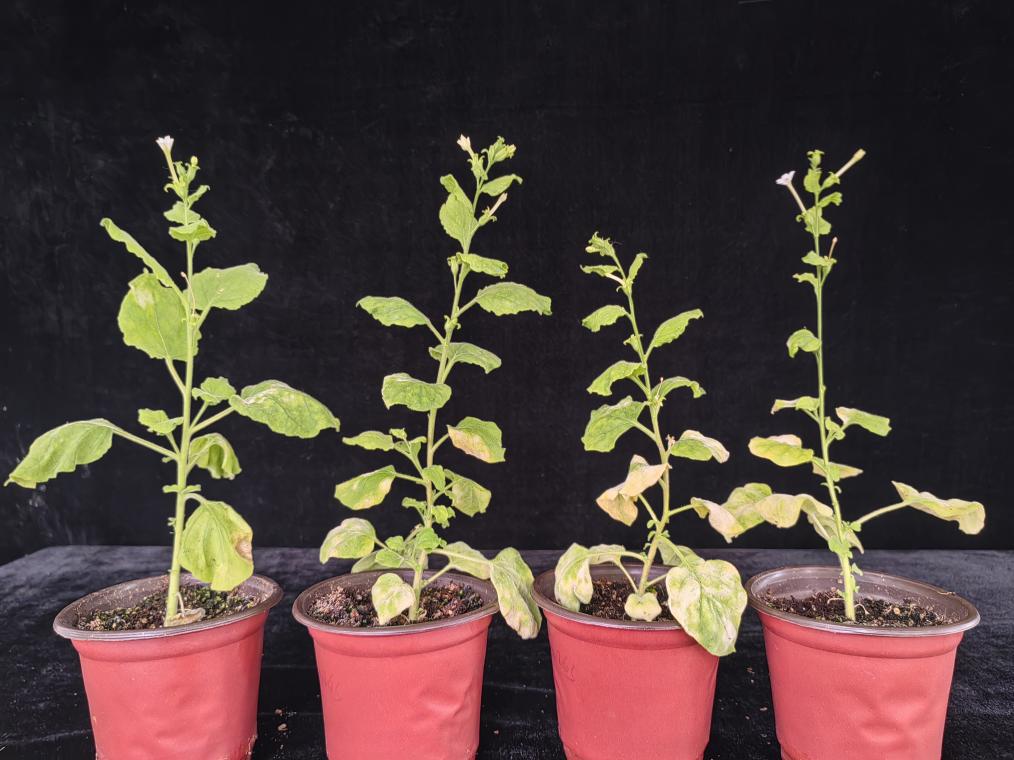


**VIGS-NtCAD1-1**

**VIGS-NtCAD1-2**

**VIGS-NtCAD2**

**pTRV2**

**empty vector**
